# Opening New Roads for Multi‐Directional Functional Applications Through In Vitro Chemical and Biological Analysis of *Zoegea leptaurea* L. Extracts

**DOI:** 10.1002/fsn3.70261

**Published:** 2025-05-13

**Authors:** Sakina Yagi, Giovanni Caprioli, Gabriele Rocchetti, Filomena Nazzaro, Florinda Fratianni, Francesca Coppola, Ozgur Yuksekdag, Ismail Koyuncu, Laura Acquaticci, Simone Angeloni, Mehmet Maruf Balos, Ulku Yerebasan, Gokhan Zengin

**Affiliations:** ^1^ Department of Botany Faculty of Science, University of Khartoum Khartoum Sudan; ^2^ Chemistry Interdisciplinary Project (ChIP), School of Pharmacy University of Camerino Camerino MC Italy; ^3^ Department of Animal Science, Food and Nutrition Università Cattolica del Sacro Cuore Piacenza Italy; ^4^ Institute of Food Science, Italian National Research Council Avellino Italy; ^5^ University Federico II of Naples Napoli Italy; ^6^ Department of Medical Biochemistry Faculty of Medicine, Harran University Sanliurfa Türkiye; ^7^ Şanlıurfa Provincial Directorate of National Education Mehmet Gunes Anatolian High School Şanlıurfa Türkiye; ^8^ Department of Pharmacology and Toxicology Bingol University, Faculty of Veterinary Medicine Bingöl Türkiye; ^9^ Physiology and Biochemistry Laboratory, Department of Biology, Science Faculty Selcuk University Konya Türkiye

**Keywords:** anti‐biofilm, antioxidant, cytotoxicity, enzyme inhibition, phenolics, probiotic, *Zoegea leptaurea* L.

## Abstract

*Zoegea leptaurea* L. has been traditionally used to treat digestive issues, headaches, and skin diseases. This study aimed to evaluate, for the first time, the phenolic content, antioxidant potential, enzyme inhibition, cytotoxic anti‐biofilm/probiotic properties of the flowers, stems, leaves, and aerial parts of *Z. leptaurea*. Results indicated that 70% EtOH extraction yielded the highest total phenolic and flavonoid contents across different plant organs, with the highest levels recorded in the flowers (44.86 mg gallic acid equivalent (GAE)/g) and leaves (28.84 mg rutin equivalent (RE)/g), respectively. Chlorogenic acid was the predominant compound in the stems and leaves, with the highest concentration obtained using 70% EtOH (5919.1 mg/kg and 10,786.70 mg/kg, respectively). The 70% EtOH extract of the flowers exhibited the strongest antiradical activity (2,2‐diphenyl‐1‐picrylhydrazyl [DPPH] = 45.10 mg trolox equivalent (TE)/g); 2,2′‐azino‐bis(3‐ethylbenzothiazoline‐6‐sulfonic acid [ABTS] = 64.53 mg TE/g) and ion‐reducing capacity (Cupric reducing antioxidant capacity [CUPRAC] = 118.81 mg TE/g; ferric reducing antioxidant power [FRAP] = 65.29 mg TE/g). The EtOH extract of the flowers and the EtOAc extract of the aerial parts exhibited the highest anti‐acetylcholinesterase activity (2.79 and 2.56 mg galantamine equivalent (GALAE)/g), with the latter also displaying the strongest anti‐butyrylcholinesterase activity (3.35 mg GALAE/g). The strongest cytotoxic effect was observed in the EtOAc extract of the leaves against lung adenocarcinoma cells (A549), with an IC_50_ value of 18.39 μg/mL. Additionally, the inhibitory activity of the extracts against immature and mature biofilms formed by pathogenic bacteria was assessed, revealing notable antibiofilm activity. Concurrently, the extracts stimulated the growth of five probiotic strains, with some reaching up to six times their respective control growth levels. In conclusion, the findings of this study suggest that *Z. leptaurea* is a promising source of bioactive compounds and warrants further investigation for its potential role in the treatment of oxidative stress‐related diseases.

## Graphical abstract

*Zoegea leptaurea* is a promising source of bioactive compounds and warrants further investigation for its potential role in the treatment of global health problems.

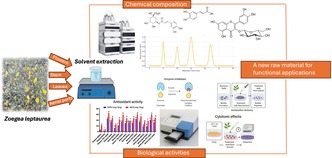

## 1 Introduction

Free radicals play an important role in different biological activities like cell signaling and destroying tumor cells. However, their excess causes harmful effects to the cell as they induce oxidative stress and consequently the development of several health disorders including Alzheimer's, cancer, diabetes, and cardiovascular diseases among other free radicals associated diseases (İzol et al. 2025; Kumar et al. 2021). Antioxidants protect cells against free radicals by inhibiting their formation or limiting their damage. Antioxidants can be produced internally by the activity of body enzymes or externally from foods containing vitamins A, E, and C, minerals, and phytochemicals among which polyphenols play a critical role (Nwozo et al. 2023).

Plants constitute an important source of health‐promoting phytochemicals with interesting biological properties like antioxidants, antimicrobial, anti‐inflammatory, anticancer, and enzyme inhibitory activities (Barreca 2020). However, despite the extensive research performed on plants, there are still a large number of them remaining unexploited for their pharmacological potential.

The genus *Zoegea* belongs to the family Asteraceae and is represented by 3 species and 6 subspecies. Members of the genus are mainly found in the Middle East and Central Asia. The Irano‐Turanian region was suggested as the main differentiation and gene center of *Zoegea* (Negaresh and Rahiminejad 2019). *Zoegea* species grow mainly in dry mountainous regions but are also found in riversides, vineyards, and shrub land. The genus comprises unarmed annual herbs with heterogamous capitula, purple‐stained phyllaries, membranous appendages, dentate or cuspidate, and strongly compressed achenes (Negaresh and Rahiminejad 2019). In Turkey, only one *Zoegea* taxon, namely, *Z. leptaurea* subsp. *leptaurea*, is reported. The plant is a perennial herb with a short stem and small white flowers. It is used traditionally to cure digestive problems, headaches, and skin conditions. It is also used as an ingredient in herbal teas. Generally, species belonging to this genus are poorly investigated. Comprehensive studies on the morphological, palynological, and anatomical characteristics of *Zoegea* species were performed by Mahmoodi et al. (2018). Only one chemical study was found reporting the isolation of 9α‐hydroxyparthenolide from the aerial parts of *Z. leptaurea* subsp. *mesopotamica* (Czerep.) Rech. (syn. *Z. mesopotamica* Czerep.) (Nawrot et al. 2015).

Considering all the aforementioned issues, the present study was designed to examine the phenolic profiles and biological activities of different extracts obtained from flowers, stems, leaves, and aerial parts of *Z. leptaurea*. Their antioxidant activity was tested by evaluating their capacity to scavenge free radicals, chelate, and reduce metal ions, while their ability to inhibit enzymes was evaluated against enzymes implicated in diabetes, skin hyperpigmentation, and Alzheimer's diseases. Additionally, a panel of cancer cells, including HCT‐116 (colorectal carcinoma), A549 (lung adenocarcinoma), HELA (cervix adenocarcinoma) and MDA‐MB‐231 (breast adenocarcinoma) cells, was used to evaluate their cytotoxicity. Finally, the extracts were examined for their anti‐biofilm effects on some pathogenic bacteria and their probiotic effects on some probiotic strains.

## 2 Materials and Methods

### 2.1 Plant Material and Extraction Methods

In 2021, a plant sample collection was conducted in Karaköprü Village, Şanlıurfa, Between Şanlıurfa and Diyarbakır (Turkey) (GPS coordinates: 37°13′45.69″ N, 38°47′42.06″ E, 615 m). Botanist Dr. Mehmet Maruf Balos carefully performed the taxonomic identification of the specimens that were obtained. For future use and confirmation, a voucher specimen (voucher number: M. Balos 5245) was formally placed at the Harran University herbarium. To maintain their phytochemical integrity, the plant's parts (flowers, leaves, stems) were meticulously separated after gathering and allowed to dry in the shade at room temperature. The plant components were uniformly combined to yield the aerial part. The dried material was then finely ground into powder using a standardized process. To prevent degradation and ensure long‐term stability, the powdered plant material was stored in light‐proof containers under controlled conditions. Four different solvents were used in the extraction process: ethanol, water, a 70% ethanol/water combination, and ethyl acetate. A 10‐g sample was macerated with 200 mL of ethanol, ethyl acetate, and an ethanol‐water mixture for 24 h at room temperature. The aqueous extract was made by infusing 10 g of plant material with hot water for 15 min. The resultant aqueous extract was then freeze‐dried after the organic solvents were eliminated using low‐pressure evaporation.

### 2.2 Assay for Total Phenolic and Flavonoid Content

Our earlier work examined total phenolics and flavonoids (Slinkard and Singleton 1977). Folin–Ciocalteu and AlCl_3_ methods were performed to determine the total phenolic and flavonoid contents in the tested extracts. Gallic acid (GA) and rutin (R) were utilized as reference standards in the experiments, and the results were presented as gallic acid equivalents (GAE) and rutin equivalents (RE).

### 2.3 Metabolomic Analysis Using LC–MS/qTOF

Metabolomic analyses were carried out utilizing an Agilent 1290 Infinity II liquid chromatography (LC) system and an Agilent 6546 LC/MS QTOF mass spectrometer (Agilent, USA). Metabolite separation was done with a Poroshell 120 EC‐C18 column (2 × 150 mm, 2.7 μm, Agilent, USA). Data S1 provide all the analytical details.

### 2.4 Assays for In Vitro Antioxidant Capacity

Antioxidant assays were performed as previously described (Grochowski et al. 2017). Trolox equivalents (TE) per gram were calculated for radical scavenging with FRAP, CUPRAC, DPPH, and ABTS. The antioxidant potential was determined in millimoles of TE per gram of extract using the phosphomolybdenum (PBD) assay, and the metal chelating activity (MCA) was quantified in EDTA.

### 2.5 Inhibitory Effects Against Some Key Enzymes

Samples were subjected to enzyme inhibition tests using the following methods (Grochowski et al. 2017): milligrams of galanthamine equivalents (GALAE) inhibited AChE and BChE, while acarbose equivalents (ACAE) per gram of extract inhibited amylase and glucosidase. Tyrosinase inhibition was measured in milligrams of kojic acid equivalents per gram of extract.

### 2.6 Cell Assays

#### 2.6.1 Cell Culture

The following cancer and normal cell lines obtained from ATCC and stored in liquid nitrogen were used for the study. HCT‐116 (Colon Cancer), A549 (Lung Cancer), HELA (Cervix Cancer), and MDA‐MB‐231 (Breast Cancer) cells were cultured in DMEM‐F12/RPMI‐1640 media supplemented with 10% fetal bovine serum (FBS), 100 μg/mL of streptomycin, and 100 IU/mL of penicillin in incubators at 37°C under humid conditions containing 5% CO_2_.

#### 2.6.2 Cell Viability Assay

For 24 h, cells from HCT‐116, A549, HELA, and MDA‐MB‐231, at 1 × 104 cells per well, were incubated in a sterile plate. The medium was taken out, and the extracts were allowed to incubate for 24 h with doses ranging from 0 to 200 μg/mL. Ten microliters of MTT (0.5 mg/mL) was added to each as a reagent. Following a 4‐h incubation, the medium was discarded and substituted with 100 μL of DMSO, after which absorbance was measured at OD570–OD690 nm utilizing a plate reader (Thermo Multiskan GO, Thermo, USA). After performing these measurements, graphs were created and the IC_50_ value was computed.

#### 2.6.3 Apoptotic Effect of the Ethyl Acetate Extract of Leaves on A549 Cancer Cells With Acridine Orange/Ethidium Bromide (AO/EB) Staining

The apoptosis of A549 cells was morphologically detected following the application of a 20 μg/mL ethyl acetate extract of leaves. The extract‐treated cells were rinsed with PBS following incubation and subsequently fixed with 70% ethanol. Following fixation, the cells were rinsed with distilled water, stained using an acridine orange/ethidium bromide working solution (Cat No./ID: A6014‐E1510) from Sigma Aldrich, Germany, and subsequently imaged under a fluorescence microscope.

#### 2.6.4 Apoptotic Effect of the Ethyl Acetate Extract of Leaves on A549 Cancer Cells With Annexin V

The Commercial FITC Annexin V Apoptosis Detection Kit I (BD Biosciences, New Jersey, USA) was utilized in accordance with the manufacturer's procedure. A549 cells were seeded in 6‐well plates at a density of 5 × 10^5^ cells per well. After 24 h of incubation, a concentration of 20 μg/mL of the ethyl acetate extract of leaves was administered, followed by an additional 24 h of incubation. Cells were cultured with trypsin and subsequently transferred to fresh 1 × 106‐inch tubes with 1X binding buffer. Each tube underwent incubation for 15 min at ambient temperature. Subsequently, 5 μL of fluorochrome‐conjugated Annexin V and 5 μL of Propidium Iodide were introduced. One hundred microliters of 1X binding buffer was introduced to the cells, followed by centrifugation at 1200 rpm and a 5‐min incubation. Ultimately, cells were examined using flow cytometry (BD, New Jersey, United States of America).

#### 2.6.5 Cell Cycle Analysis of the Ethyl Acetate Extract of Leaves on A549 Cancer Cell

We utilized the BD Cycle‐test kit (New Jersey, USA) incorporating PI dye to examine the cell cycle. The protocol entailed inoculating 1 × 10^6^ cells/mL into sterile 6‐well plates and administering the specified dose of the ethyl acetate leaf extract (20 μg/mL) roughly 1 day thereafter. Cells were incubated for an additional 24 h. Cells were detached using trypsin and centrifuged at 1500 rpm for 5 min; subsequently, the supernatant was discarded and the pellet was resuspended. One milliliters of PBS was introduced to the cell suspensions and subjected to two wash cycles. One volume of binding buffer (for dye binding) was introduced, and the supernatant was eliminated using centrifugation at 1500 rpm for 5 min. Two hundred fifty microliters of solution A (Trypsin) was added to the cell pellet and incubated in the dark for 10 min with gentle agitation. Subsequently, 200 μL of solution B (Trypsin inhibitor) was introduced and incubated in the dark for 10 min. Cells were treated with 200 μL of solution C (PI) for 10 min in darkness and subsequently analyzed using a BD Facs flow cytometry equipment (BD, New Jersey, USA).

### 2.7 Antibiofilm Activity of the Extracts

The effect of the extracts of the different parts of *Z*. *leptaurea* on immature and mature biofilm was assessed using 96‐well flat‐bottomed microtiter plates (Falcon, VWR International, Milan, Italy) (Zengin et al. 2024). Overnight bacterial cultures, standardized to 0.5 McFarland using fresh culture broth, were diluted, and 10 μL of this suspension was added to each well. For the evaluation of the effect of the extracts on the immature biofilm, they were introduced at a concentration of 20 mg/mL, along with sterile Luria‐Bertani (LB) broth (Sigma Aldrich Italy, Milan, Italy), bringing the total volume to 250 μL per well. The plates were sealed with parafilm tape to prevent evaporation and incubated at 37°C (or 35°C for specific bacterial strains) for 48 h. For the evaluation of the effect of the extracts on mature biofilm, after 24 h of bacterial growth, the supernatant was discarded and substituted with fresh LB broth and were added at a concentration of 20 mg/mL, bringing the total volume to 250 μL per well, and the plates were incubated for another 24 h. Following the total 48 h incubation, non‐adherent planktonic cells were carefully removed, and the wells were gently rinsed twice with sterile phosphate‐buffered saline (PBS). To fix the remaining sessile cells, 200 μL of methanol was added for 15 min and then discarded. Once the wells had dried, the biofilms were stained with 200 μL of 2% (w/v) crystal violet solution for 20 min. After removing excess dye, the wells were gently washed with sterile PBS and left to dry. The bound dye was then released by adding 200 μL of 20% (w/v) glacial acetic acid, and absorbance was measured at 540 nm using a Cary 50 Bio spectrophotometer (Varian). The percentage of biofilm inhibition was calculated relative to the untreated control, where bacterial growth was considered uninhibited. All experiments were conducted in triplicate, and the results were expressed as mean ± standard deviation (SD).

### 2.8 Assessment of *Z. leptaurea* Extracts Impact on Sessile Cell Metabolism

To evaluate the effect of the extracts (5 mg/mL) on the metabolic activity of sessile bacterial cells, an MTT colorimetric assay was performed (Francolino et al. 2025). The extracts were added either at the beginning of bacterial growth or after 24 h to assess their influence on metabolic activity over time. After a total incubation period of 48 h, non‐adherent planktonic cells were removed, and 150 μL of PBS along with 30 μL of 0.3% MTT solution (Sigma, Milan, Italy) was introduced into each well. The plates were incubated for 2 h at 37°C (or 35°C depending on the bacterial strain). Following incubation, the MTT solution was discarded, and the wells were washed twice with 200 μL of sterile PBS. The resulting formazan crystals were then dissolved by adding 200 μL of DMSO, and absorbance was measured at 570 nm using a Cary 50 Bio spectrophotometer (Varian).

### 2.9 Assessment of *Z. leptaurea* Extracts on Probiotics Growth

*Lactobacillus bulgaricus* , *Lactocaseobacillus casei* Shirota (LcS), *Lactobacillus gasseri* LG050, *Lactiplantibacillus plantarum* 299 V, and *Lacticaseibacillus rhamnosus* GG were obtained from a commercial formulation available in a local pharmacy. *Lactobacillus bulgaricus* (DSM 20081) was provided by DMSZ (Braunschweig‐Süd, Germany).

### 2.10 Growth of Lactic Acid Bacteria in the Presence of the Extracts

The bacterial strains were incubated at 37°C, except for *L. plantarum* , which was cultured at 30°C for 16–18 h in MRS broth (Liofilchem, Roseto degli Abruzzi, Italy) supplemented with 20 mg/mL of *Z. leptaurea* extracts (Nazzaro et al. 2024). Bacterial growth was measured at a wavelength of 600 nm (Cary 50 Bio, Varian, Palo Alto, CA, USA). The impact of the five types of honey on lactic acid bacteria growth was expressed as a percentage relative to the control, where strains were grown in conventional MRS (without the addition of extracts), for which we assumed a percentage of growth = 100.

### 2.11 Statistical Analysis

All the experiments were carried out in triplicate, and statistical comparisons among extract groups were made using a one‐way ANOVA followed by Tukey's post hoc multiple comparison test. Statistical analyses were carried out with GraphPad Prism (version 9.2), and *p* < 0.05 was statistically significant.

Pearson's correlation coefficients were calculated using PASW Statistics 26.0 (SPSS Inc., Chicago, IL. USA) to highlight significant correlations (*p* < 0.01 and *p* < 0.05; two‐tailed) between the phytochemical contents (TPC and TFC) and in vitro antioxidant and enzymatic activities. Also, the concentration of each phenolic compound in the different plant extracts was loaded in the software MetaboAnalyst 6.0 to perform unsupervised hierarchical clustering analysis, then inspecting the different sample grouping and up‐ versus down‐accumulation of the quantified compounds.

## 3 Results and Discussion

### 3.1 Total Phenolic (TPC) and Flavonoids (TFC) Contents

**FIGURE 1:**
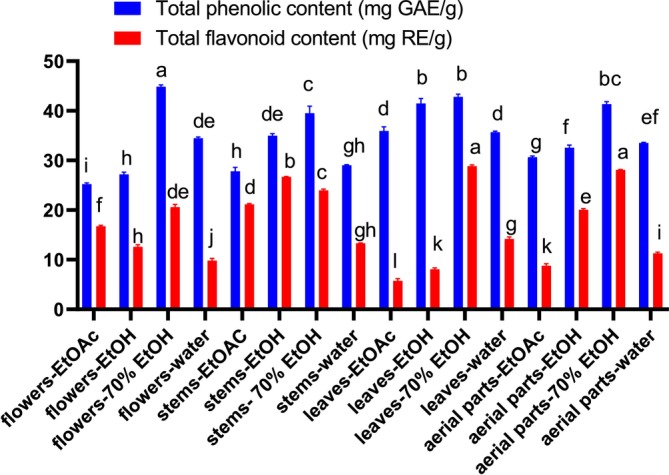
Total phenolic and flavonoid contents in extracts of *Zoegea leptaurea*. Values are reported as mean ± SD of three parallel measurements. GAE, gallic acid equivalents; RE, rutin equivalents. Different letters in the same assay indicate significant differences between the tested extracts (*p* < 0.05).

The therapeutic effectiveness of polyphenolics is always associated with their strong antioxidant properties, which can effectively attenuate oxidative stress by scavenging free radicals (Mugundhan et al. 2024). In the present study, the TPC and TFC in extracts from different organs of *Z. leptaurea* were determined, and results are presented in Figure 1. The TPC was in the range of 25.24–44.86 mg GAE/g, with the 70% EtOH extract of the flower displaying the highest significant (*p* < 0.05) content. In fact, it was observed that 70% EtOH was the best solvent to recover the highest TPC in all organs, in addition to EtOH of the leaves. The TFC was in the range of 5.72–28.84 mg RE/g, with the 70% EtOH extract of the leaves and aerial parts recording the highest content (*p* ≥ 0.05). It was noted that the EtOAc, EtOH, and 70% EtOH extracts of the stem also accumulated high TFC (21.16, 26.67 and 23.98 mg RE/g respectively, *p* < 0.05). Overall, the results indicated that *Z. leptaurea* is rich in phenolics, and they were highly recovered by 70% EtOH.

### 3.2 The Chemical Profile of *Z. leptaurea* Extracts Is Affected by Extraction Solvents

The presence and quantity of selected standard phenolic compounds in different extracts of the flowers, stem, leaves, and aerial parts were examined, and results are presented in Tables 1, 2, 3, 4. From the 38 standards used, 16 were detected and the total concentrations of all compounds in extracts of flower, leaves, and aerial parts were in the following order: 70% EtOH > EtOH > H_2_O > EtOAc, while in the stem, it was as follows: EtOH > 70% EtOH > H_2_O > EtOAc. Chlorogenic acid was the major compound in the stems (EtOH = 5533.36, 70% EtOH = 5919.1 and H_2_O = 4530.09 mg/kg) and leaves (EtOH = 1752.64, 70% EtOH = 10,786.70 and H_2_O = 8458.80 mg/kg). Caffeic acid (EtOH = 3570.00 mg/kg; 70% EtOH = 4975.00 mg/kg) and chlorogenic acid (70% EtOH = 2883.69; H_2_O = 2228.51 mg/kg) were the principal compounds in the extracts of the flowers. Although not detected in the flowers, stem, and leaves extracts, catechin was the major compound in EtOH (5074.92 mg/kg), 70% EtOH (26,348.65 mg/kg), and aqueous (19,519.59 mg/kg) extracts of the aerial parts. Also, procyanidin B2 was only detected in the aerial parts extracts (276.69–296.91 mg/kg).

**TABLE 1:** Chemical composition of flower extracts of *Zoegea leptaurea*.

Compounds	EtOAc	EtOH	70% EtOH	Water
Gallic acid	4.59	4.47	20.45	7.85
Neochlorogenic acid	35.47	357.03	1775.13	1761.66
Chlorogenic acid	81.85	485.65	2883.69	2228.51
4‐Hydroxy benzoic acid	84.37	94.12	146.91	137.25
Caffeic acid	nd	3570.00	4975.00	137.24
Vanillic acid	nd	nd	213.20	nd
Syringic acid	nd	nd	145.16	nd
P‐Coumaric acid	10.03	11.70	38.29	38.64
Ferulic acid	57.99	57.25	134.95	150.13
Rutin	4.54	9.07	17.05	10.86
Isoquercitrin	32.75	200.74	1101.50	459.01
Delphindin 3,5 diglucoside	48.33	239.90	1111.62	527.59
Phloridzin	1.19	nd	nd	nd
Kaempferol‐3‐glucoside	306.80	554.53	2836.11	1467.31
Ellagic acid	nd	nd	116.33	nd
Quercetin	nd	nd	129.13	12.17
Isorhamnetin	8.13	7.64	70.16	15.25
Total amounts	676.04	5592.09	15,714.69	6953.46

Abbreviation: nd, not detected.

**TABLE 2:** Chemical composition of stem extracts of *Zoegea leptaurea*. nd = not detected.

Compounds	EtOAc	EtOH	70% EtOH	Water
Gallic acid	8.00	18.41	15.51	8.63
Neochlorogenic acid	15.49	1366.03	1374.43	1754.52
Chlorogenic acid	75.78	5533.36	5919.16	4530.09
4‐Hydroxy benzoic acid	87.83	136.96	118.49	115.79
Caffeic acid	45.52	91.77	83.25	80.32
Vanillic acid	nd	nd	234.54	286.56
Syringic acid	nd	150.48	147.4	150.33
P‐Coumaric acid	15.38	35.19	28.53	30.32
Ferulic acid	60.64	95.94	100.32	112.75
Rutin	4.29	44.40	38.29	25.64
Isoquercitrin	24.95	539.11	619.86	260.38
Delphindin 3,5 diglucoside	27.99	609.60	690.66	286.15
Kaempferol‐3‐glucoside	236.74	1739.43	nd	869.76
Ellagic acid	nd	25,13	32,32	nd
Quercetin	nd	24,89	28,21	nd
Isorhamnetin	9.77	43.95	44.31	8.52
Total amounts	**612.37**	**10457.21**	**9475.33**	**8519.77**

**TABLE 3:** Chemical composition of leaves extracts of *Zoegea leptaurea*.

Compounds	EtOAc	EtOH	70% EtOH	Water
Gallic acid	nd	nd	7.56	7.00
Neochlorogenic acid	nd	366.32	1124.34	1385.82
Chlorogenic acid	42.85	1752.64	10,786.70	8458.80
4‐Hydroxy benzoic acid	102.49	107.19	109.98	109.82
P‐Coumaric acid	11.88	14.66	18.68	17.74
Ferulic acid	nd	nd	92.55	86.64
Rutin	nd	10.90	16.50	10.25
Isoquercitrin	4.92	138.28	306.12	114.31
Delphindin 3,5 diglucoside	nd	162.84	338.58	126.37
Kaempferol‐3‐glucoside	119.50	585.22	1528.96	556.67
Total amounts	281.64	3138.05	14,339.09	10,873.42

Abbreviation: nd, not detected.

**TABLE 4:** Chemical composition of aerial part extracts of *Zoegea leptaurea*.

Compounds	EtOAc	EtOH	70% EtOH	Water
Gallic acid	nd	7.94	9.07	7.47
Neochlorogenic acid	23.16	456.39	1332.93	1752.47
Catechin	nd	5074.92	26,348.65	19,519.59
Procyanidin B2	nd	296.91	285.99	276.69
Chlorogenic acid	132.12	nd	nd	nd
4‐Hydroxy benzoic acid	112.33	nd	nd	nd
Caffeic acid	57.24	80.49	86.77	82.66
Vanillic acid	nd	210.81	nd	227.70
Syringic acid	nd	nd	nd	142.22
P‐Coumaric acid	17.18	24.67	25.63	25.79
Ferulic acid	78.46	84.82	100.81	100.13
Rutin	nd	16.83	294.47	13.52
Isoquercitrin	27.87	302.76	497.42	194.91
Delphindin 3,5 diglucoside	37.56	344.72	575.40	233.71
Kaempferol‐3‐glucoside	283.58	nd	2025.05	854.41
Ellagic acid	nd	nd	34.91	nd
Quercetin	5.10	11.38	33.97	nd
Isorhamnetin	nd	nd	25.03	6,61
Total amounts	774.60	6912.61	31,676.10	23,437.86

Abbreviation: nd, not detected.

**FIGURE 2:**
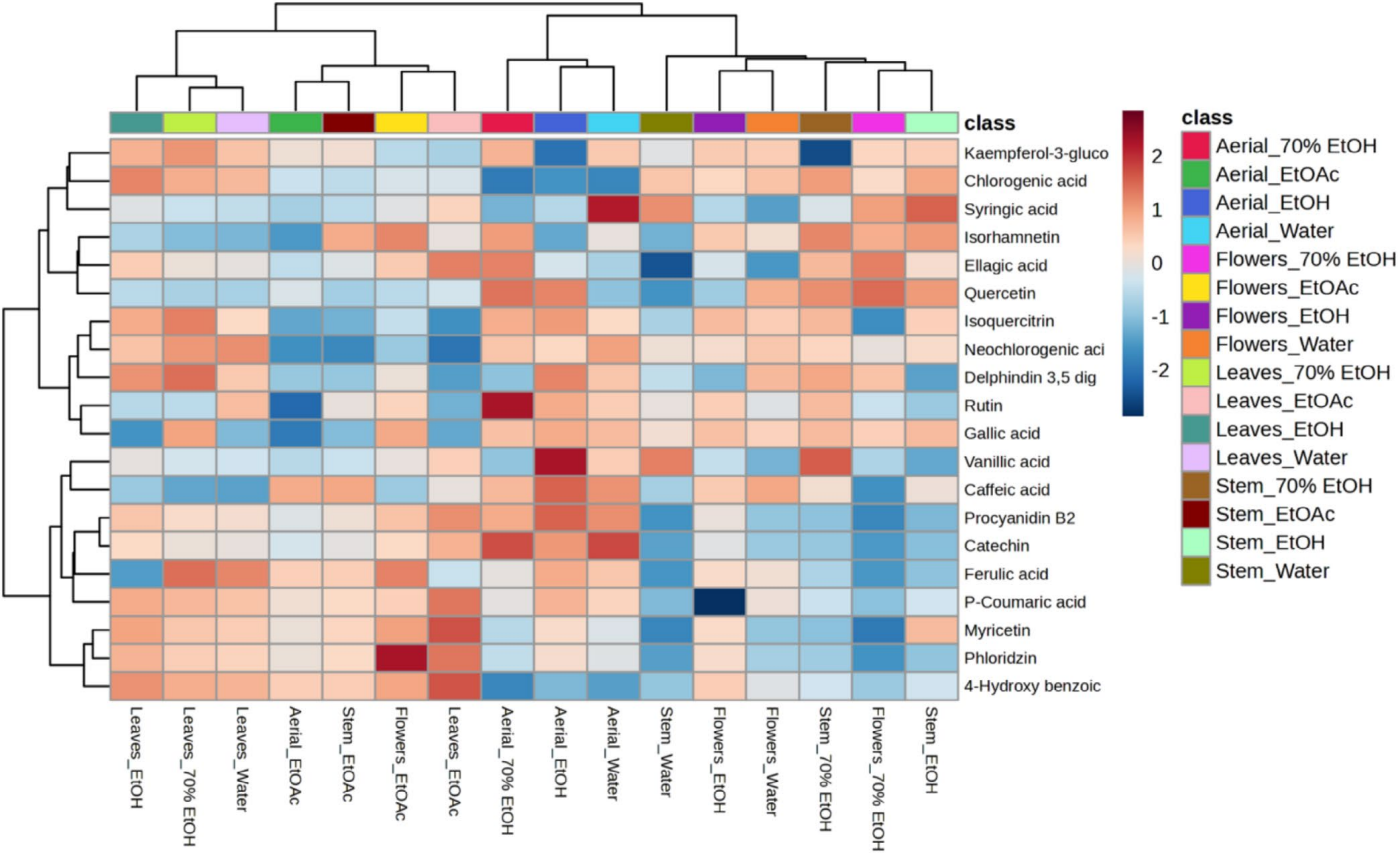
Heat map resulting from the unsupervised hierarchical cluster analysis showing the up‐ and down‐accumulation of the main phenolic compounds characterizing each extract under investigation.

EtOAc extract of the four organs was dominated by kaempferol‐3‐glucoside (119.50–306.80 mg/kg). Neochlorogenic acid was found in relatively considerable amounts in the EtOH (366.32–1366.03 mg/kg), 70% EtOH (1124.34–1775.13 mg/kg), and aqueous (1385.82–1761.66 mg/kg) extracts of the four organs. Isoquercitrin and delphinidin 3,5 diglucoside were also detected in the extracts of the four organs in relatively considerable amounts with the highest accumulation in the 70% EtOH extract of the flower (1101.50 and 1111.62 mg/kg respectively). Other compounds like 4‐hydroxybenzoic acid, ferulic acid, p‐coumaric acid, syringic acid, vanillic acid, rutin, ellagic acid, and isorhamnetin were also detected in most extracts but in lesser content. Thus, it was clear that this plant could be a new source of phenolic compounds. Interestingly, the unsupervised hierarchical heat map built considering the concentration of each detected phenolic compound in the different plant parts showed intriguing results (Figure 2). Particularly, according to the phenolic profile detected, we found two main groups; the first one hierarchically discriminated the EtOAc extracts together with leaf extracts, while the second one included all the remaining samples. Therefore, the unsupervised statistics demonstrated the pivotal role of each different solvent to promote the recovery of the antioxidant phenolic compounds.

### 3.3 In Vitro Antioxidant Activity

**FIGURE 3:**
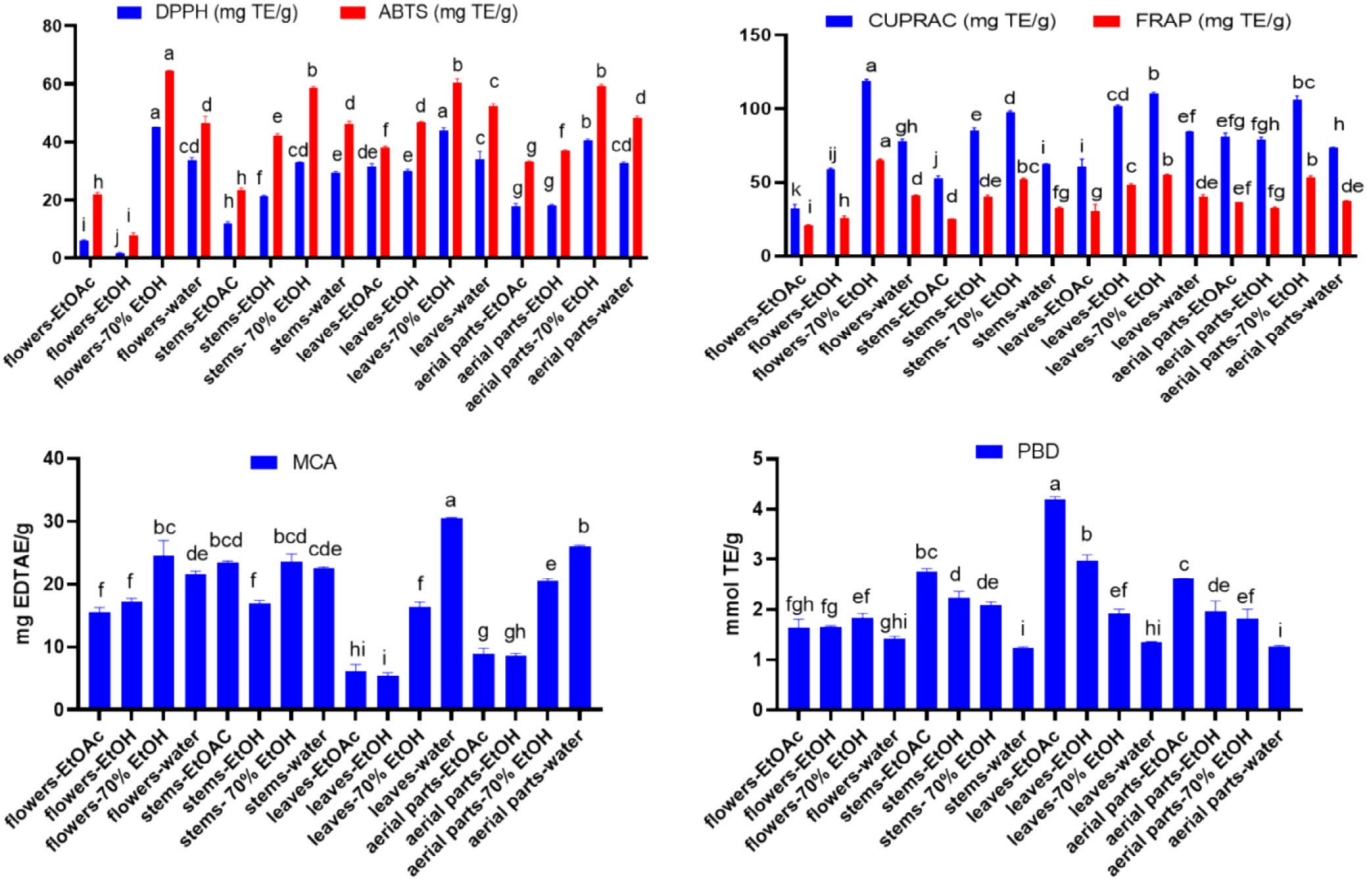
Antioxidant properties of extracts of *Zoegea leptaurea*. Values are reported as mean ± SD of three parallel measurements. EDTAE, EDTA equivalent; MCA, metal chelating activity; PBD, phosphomolybdenum; TE, trolox equivalent. Different letters in the same assay indicate significant differences between the tested extracts (*p* < 0.05).

Oxidative stress is associated with serious health problems like cardiovascular, cancer, and neurodegenerative diseases. Antioxidants counteract ROS and hence there is a continuous demand to explore new natural sources of antioxidants (Kumar et al. 2021). Extracts from different organs of *Z. leptaurea* were evaluated for their antioxidant property by DPPH, ABTS, CUPRAC, FRAP, MCA, and PBD assays. Results are presented in Figure 3. The DPPH radical scavenging activity was in the range of 1.69–45.10 mg TE/g with the highest effect exerted by the 70% EtOH extract of the flowers and leaves (*p* ≥ 0.05). The ABTS radical scavenging activity was in the range of 7.96–64.53 mg TE/g with the highest effect displayed by the 70% EtOH extract of the flower followed by that of the stem, leaves, and aerial parts (58.76–60.40 mg TE/g, *p* ≥ 0.05). The Cu^++^ and Fe^+++^ reducing capacity of the extracts was in the range of 32.37–118.81 and 20.84–65.29 mg TE/g, respectively, with the highest effect recorded from the 70% EtOH extract of the flower followed, respectively, by that of the leaves and aerial parts. Concerning the chelating property of extracts, it was in the range of 5.37–32.52 mg EDTAE/g with the best effect exhibited by the aqueous extract of the leaves and aerial parts, respectively (*p* < 0.05). The total antioxidant activity of extracts was in the range of 1.23–4.19 mmol TE/g with the EtOAc extract of the leaves recording the highest significant activity (*p* < 0.05).

Overall, all investigated organs displayed promising antioxidant activity, and 70% EtOH was the best solvent to recover molecules with the best anti‐radical and reducing ions activity. Earlier studies have shown a positive correlation between the phenolic content of extracts of various plants and their antioxidant activity (Nwozo et al. 2023; Sytar et al. 2022). In the present study, extracts accumulated high content of some phenolic compounds known for their potent antioxidant activity. The leaves, flowers, and stems had a high concentration of chlorogenic acid, which proved to exhibit remarkable antioxidant activity (Huang et al. 2023). On the other hand, 70% EtOH, EtOH, and aqueous extracts of aerial parts were dominated by the high antioxidant compound catechin (Munteanu and Apetrei 2022). Other antioxidant compounds included neochlorogenic acid (Caracciolo et al. 2016), 4‐hydroxy benzoic acid (Hurtado‐Barroso et al. 2019), isoquercitrin (Li et al. 2016), kaempferol‐3‐glucoside (Taiwo et al. 2019), caffeic acid (Sato et al. 2011), ferulic acid (Zduńska et al. 2018), vanillic acid (Surya et al. 2023), *p*‐coumaric acid (Kiliç and Yeşiloğlu 2013), and rutin (Choi et al. 2021) were also found relatively in considerable content in most extracts of the four organs. Thus, these findings suggested that *Z. leptaurea* is a promising source of antioxidant molecules for different applications.

Pearson's correlation coefficients were calculated to evaluate the ability of TPC and TFC to explain the measured bioactive properties. The results are represented for each plant part in Tables 5, 6, 7, 8. Interestingly, as far as the antioxidant activity values are concerned, TPC in flower extracts was significantly (*p* < 0.01) correlated with DPPH, CUPRAC, ABTS, FRAP, and MCA; the highest correlation coefficient was detected with FRAP activity (0.998). Conversely, the TFC detected in flower extracts showed only a significant correlation with PDB values (0.779), thus revealing that other phenolic classes (such as phenolic acids and lower‐molecular‐weight compounds) contribute to the in vitro antioxidant mechanisms. Regarding stem extracts, our findings showed that TPC was highly correlated with CUPRAC values (0.980), followed by FRAP (0.967). For these extracts, TFC showed significant correlations with CUPRAC and PDB. A very interesting finding was obtained when inspecting the correlations between TFC and antioxidant assays for leaf extracts; particularly, it was clear that the TFC was more important than TPC in explaining the bioactivity of the extracts, recording significant correlations with DPPH, CUPRAC, ABTS, MCA, and PDB, with the highest correlation coefficient observed for DPPH (0.957; *p* < 0.01). Finally, similar findings were obtained for the aerial part extracts, with both TPC and TFC being highly correlated with CUPRAC, ABTS, and FRAP; however, for these extracts, the DPPH was exclusively explained by the TPC (0.881; *p* < 0.01).

**TABLE 5:** Pearson's correlation coefficients between total bioactive contents and bioactive properties of flower extracts.

	TPC	TFC
DPPH	0.945**	ns
CUPRAC	0.975**	ns
ABTS	0.929**	ns
FRAP	0.998**	ns
MCA	0.938**	ns
PBD	ns	0.779**
AChE	ns	ns
BChE	−0.630*	ns
Tyrosinase	ns	0.762**
Amylase	ns	ns
Glucosidase	ns	ns

Abbreviation: ns, not significant (*p* > 0.05).

* *p* < 0.05.

** *p* < 0.01.

**TABLE 6:** Pearson's correlation coefficients between total bioactive contents and bioactive properties of stem extracts.

	TPC	TFC
DPPH	0.612*	ns
CUPRAC	0.980**	0.619*
ABTS	0.782**	ns
FRAP	0.967**	ns
MCA	ns	ns
PBD	ns	0.688*
AChE	ns	0.953**
BChE	ns	0.779**
Tyrosinase	ns	0.959**
Amylase	ns	0.739**
Glucosidase	0.744**	0.787**

Abbreviation: ns, not significant (*p* > 0.05).

* *p* < 0.05.

** *p* < 0.01.

**TABLE 7:** Pearson's correlation coefficients between total bioactive contents and bioactive properties of leaf extracts.

	TPC	TFC
DPPH	ns	0.957**
CUPRAC	0.874**	0.692*
ABTS	ns	0.930**
FRAP	0.881**	0.746**
MCA	ns	ns
PBD	ns	−0.657*
AChE	ns	ns
BChE	0.647*	ns
Tyrosinase	ns	ns
Amylase	ns	ns
Glucosidase	0.909**	ns

Abbreviation: ns, not significant (*p* > 0.05).

* *p* < 0.05.

** *p* < 0.01.

**TABLE 8:** Pearson's correlation coefficients between total bioactive contents and bioactive properties of aerial part extracts.

	TPC	TFC
DPPH	0.881**	ns
CUPRAC	0.887**	0.807**
ABTS	0.931**	0.678*
FRAP	0.933**	0.703*
MCA	ns	ns
PBD	ns	ns
AChE	ns	ns
BChE	ns	ns
Tyrosinase	ns	ns
Amylase	ns	ns
Glucosidase	ns	ns

Abbreviation: ns, not significant (*p* > 0.05).

* *p* < 0.05.

** *p* < 0.01.

### 3.4 Enzyme Inhibitory Activity

Natural substances from plants with the capacity to inhibit enzymes were proven to play a potential therapeutic role in the treatment of several diseases (Naz et al. 2022). In the present study, the enzyme inhibitory activity of different extracts of *Z. leptaurea* was determined against acetylcholinesterase (AChE), butyrylcholinesterase (BChE), tyrosinase (Tyr), ⍺‐amylase, and ⍺‐glucosidase, and results are presented in Table 9. The anti‐AChE activity ranged between not active and 2.79 mg GALAE/g with the highest activity recorded from the EtOH extract of the flowers and the EtOAc extract of aerial parts (2.56 mg GALAE/). The latter also exerted the highest anti‐BChE activity (3.35 mg GALAE/g) followed, respectively, by the EtOH extract of the leaves (3.19 mg GALAE/) and aerial parts (3.02 mg GALAE/). All aqueous extracts did not inhibit the two cholinesterase enzymes. The stem and flowers exhibited relatively higher anti‐Tyr activity than the leaves and the aerial parts, and the highest effect was displayed by the EtOH extract of the stem (51.2 mg KAE/g) followed, respectively, by its 70% EtOH (46.53 mg KAE/g) and EtOAc (44.26 mg KAE/g) extracts. The three organic extracts of the flowers revealed comparable anti‐Tyr activity (40.55–43.90 mg KAE/g, *p* ≥ 0.05). Concerning the two enzymes associated with the breakdown of carbohydrates, all extracts were more effective against the ⍺‐glucosidase enzyme than the ⍺‐amylase with the best activity recorded from the EtOH extract of the leaves (4.55 mmol ACAE/g) followed, respectively, by that of the stem and flowers (3.65 and 3.62 mmol ACAE/g; *p* ≥ 0.05) and the EtOAc extract of the aerial parts and 70% EtOH extract of the leaves (3.27 and 3.26 mmol ACAE/g; *p* ≥ 0.05). On the other hand, extracts revealed low enzyme inhibition against the ⍺‐glucosidase (0.07–0.75 mmol ACAE/g). Previous studies revealed that some of the identified compounds in different extracts had interesting enzyme inhibition activity. For example, ferulic acid was shown to inhibit in a concentration‐dependent manner the AChE (Mugundhan et al. 2024) and tyrosinase (Alifah et al. 2024). Also, caffeic acid and chlorogenic acid inhibited the AChE and BChE in a dose‐dependent manner, but their combination exerted antagonistic effects (Oboh et al. 2013). p‐Coumaric acid was found to be a potent tyrosinase inhibitor, even more than the well‐known inhibitors arbutin and kojic acid (Boo 2019). Caffeic acid lowered glucose levels in diabetic rats (Hsu et al. 2000). Thus, the results obtained showed that *Z. leptaurea* is a promising source of bioactive molecules for the management of diabetes, skin, and neurodegenerative diseases.

**TABLE 9:** Enzyme inhibitory effects of extracts of *Zoegea leptaurea* **.

Parts	Solvents	AChE (mg GALAE/g)	BChE (mg GALAE/g)	Tyrosinase (mg KAE/g)	Amylase (mmol ACAE/g)	Glucosidase (mmol ACAE/g)
Flowers	EtOAc	1.89 ± 0.22^e^	2.14 ± 0.24^def^	40.73 ± 1.25^c^	0.69 ± 0.03^b^	na
	EtOH	2.79 ± 0.09^a^	2.79 ± 0.20^abcd^	40.55 ± 0.70^c^	0.75 ± 0.02^a^	3.62 ± 0.39^ab^
	70% EtOH	2.00 ± 0.01^cde^	0.97 ± 0.10^g^	43.90 ± 1.25^bc^	0.43 ± 0.01^e^	1.46 ± 0.02^d^
	Water	na	na	na	0.07 ± 0.01^f^	0.29 ± 0.03^e^
Stem	EtOAc	1.98 ± 0.29^de^	2.48 ± 0.47^bcd^	44.26 ± 0.94^bc^	0.73 ± 0.02^ab^	na
	EtOH	2.38 ± 0.01^abcd^	2.35 ± 0.21^cde^	51.24 ± 0.62^a^	0.54 ± 0.02^c^	3.65 ± 0.70^ab^
	70% EtOH	1.87 ± 0.10^e^	1.32 ± 0.07^g^	46.53 ± 0.40^b^	0.48 ± 0.01^de^	2.17 ± 0.12^cd^
	Water	na	na	na	0.07 ± 0.01^f^	0.17 ± 0.02^e^
Leaves	EtOAc	2.17 ± 0.18^bcde^	1.45 ± 0.18^fg^	30.99 ± 4.03^de^	0.60 ± 0.02^c^	na
	EtOH	2.44 ± 0.13^abc^	3.19 ± 0.40^ab^	29.45 ± 0.87^ef^	0.54 ± 0.03^c^	4.55 ± 0.13^a^
	70% EtOH	1.99 ± 0.38^cde^	1.64 ± 0.19^efg^	30.70 ± 0.03^de^	0.44 ± 0.02^de^	3.26 ± 0.33^b^
	Water	na	na	na	0.07 ± 0.01^f^	0.36 ± 0.01^e^
Aerial parts	EtOAc	2.56 ± 0.10^ab^	3.35 ± 0.12^a^	30.00 ± 0.99e^f^	0.58 ± 0.02^c^	3.27 ± 0.80^b^
	EtOH	2.18 ± 0.05^bcde^	3.02 ± 0.54a^bc^	34.30 ± 0.87^d^	0.49 ± 0.02^d^	2.84 ± 0.09^bc^
	70% EtOH	1.98 ± 0.04^de^	1.39 ± 0.16^g^	26.78 ± 1.24^f^	0.46 ± 0.03^be^	1.95 ± 0.79^cd^
	Water	na	na	na	0.08 ± 0.01^f^	0.08 ± 0.03^e^

Abbreviations: ACAE, acarbose equivalent; GALAE, galantamine equivalent; KAE, kojic acid equivalent; na, not active.

** Values are reported as mean ± SD of three parallel measurements. Different letters in the same assay indicate significant differences between the tested extracts (*p *< 0.05).

Pearson's correlation coefficients were calculated to evaluate the ability of TPC and TFC to explain the measured enzymatic properties. The results are represented for each plant part in Tables 5, 6, 7, 8. Overall, significant and positive correlation coefficients were obtained in flower extracts when comparing TFC with PBD (0.779) and tyrosinase (0.762). A very important finding was observed for stem extracts, with TFC showing all significant correlations with the enzymatic activities under investigation. Particularly, the highest correlation coefficients were found with tyrosinase and AChE, being 0.959 and 0.953, respectively. As far as leaf extracts are concerned, we found a highly significant correlation between TPC and glucosidase inhibition (0.909). Finally, no significant correlations were observed for the aerial part extracts.

### 3.5 Evaluation of Cytotoxicity

**FIGURE 4:**
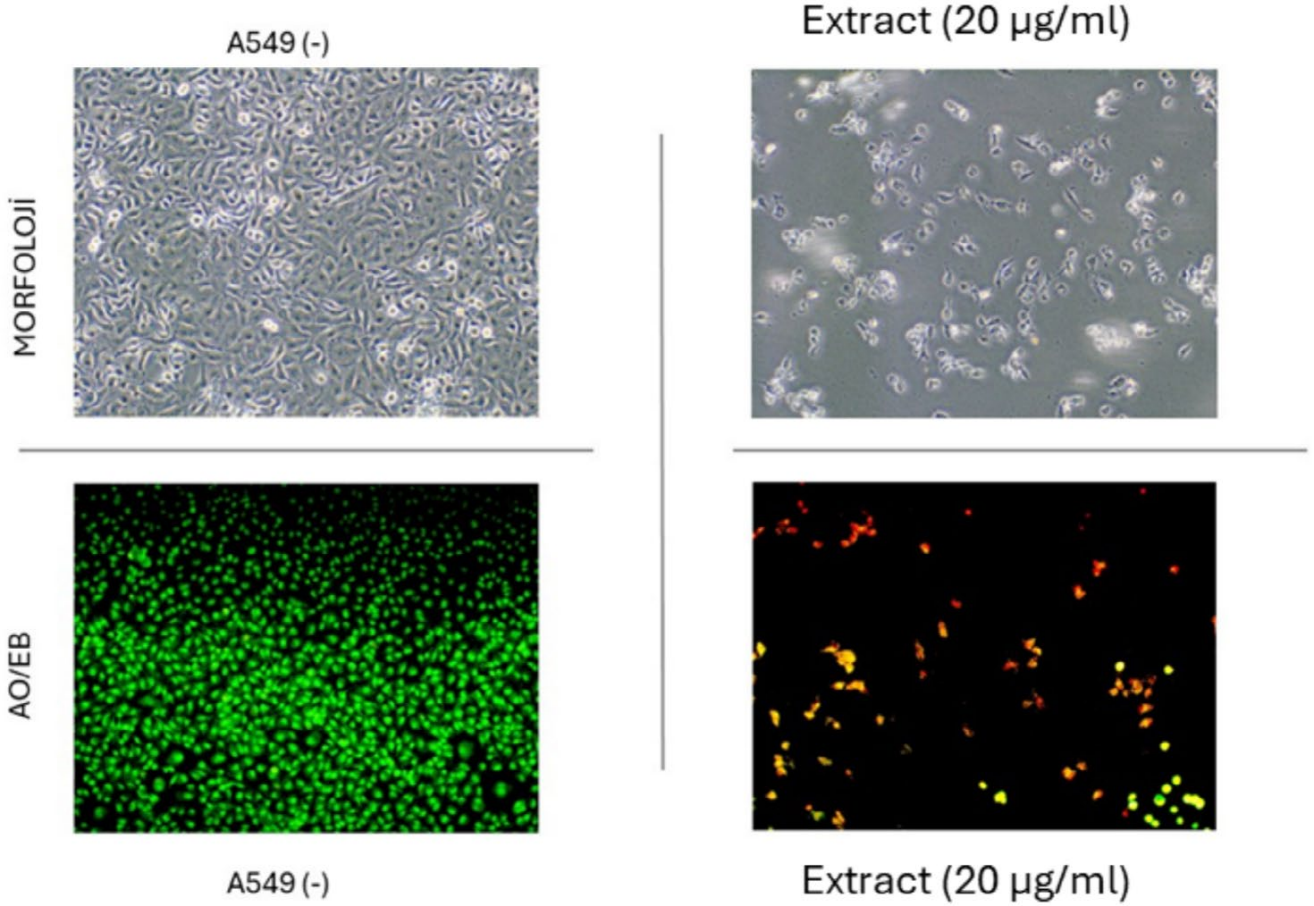
AO/EB staining after application of the ethyl acetate extract of leaves (120 μg/mL) to A549 cells (20 μg/mL) applied to A549 cells.

**FIGURE 5:**
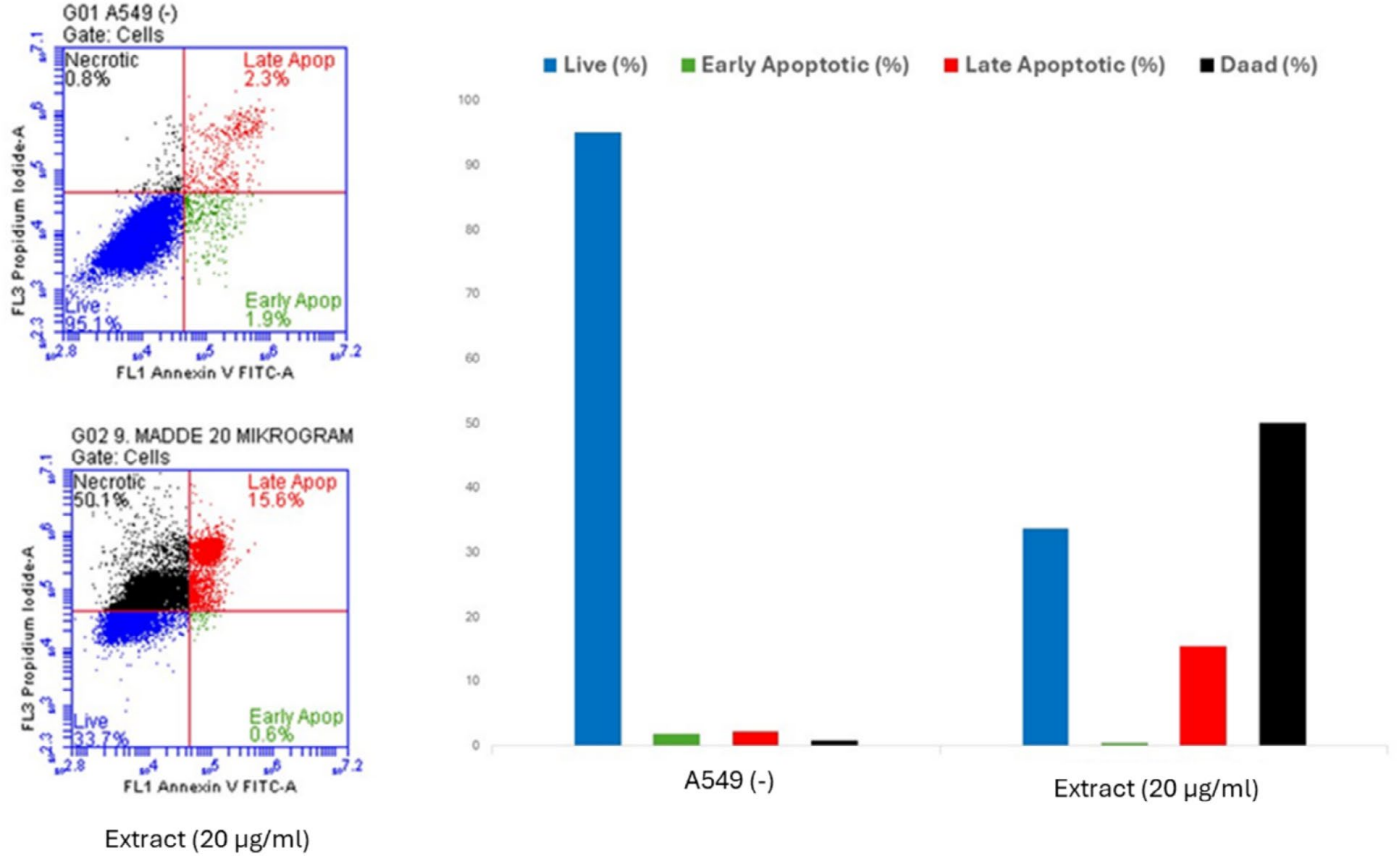
Annexin‐V/PI staining results after applying the ethyl acetate extract of leaves (20 μg/mL) to A549 cell. Blue: Live‐[(FITC−)/(PI−)]; Green: Early apoptotic [(FITC+)/(PI−)]; Red: Dead [(FITC+)/(PI+)]; Black: Necrotic [(FITC+)/(PI+)] cells.

**FIGURE 6:**
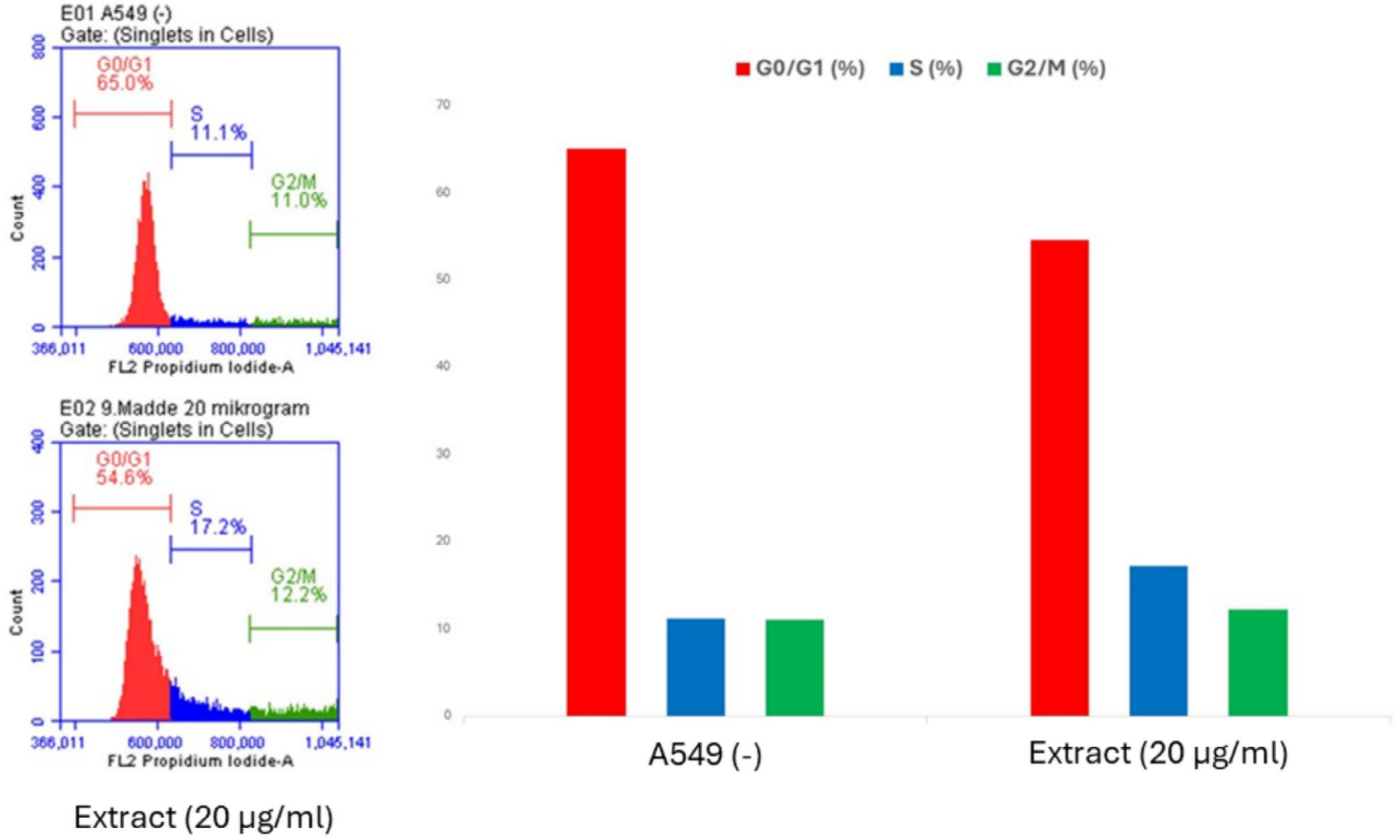
Cell Cycle staining results after applying the ethyl acetate extract of leaves (20 μg/mL) to A549 cell. Red: G0/G1 phase; Blue: S phase; Green: G2/M phase.

Cancer is considered one of the top leading causes of death in the world. Plants possess a huge reservoir of bioactive molecules that play a pivotal role in the discovery of novel substances useful for the treatment of cancer (Welz et al. 2018). In the present study, the cytotoxic effect of different extracts from *Z. leptaurea* was evaluated against the HCT‐116 (colorectal carcinoma), A549 (lung adenocarcinoma), HELA (cervix adenocarcinoma) and MDA‐MB‐231 (breast adenocarcinoma) cells, and results are depicted in Table 10. Extracts of different organs had a variable effect toward the tested cancer cells. The best cytotoxic effect was observed from the EtOAc extract of the leaves against the A549 with IC_50_ 18.39 μg/mL. Also, its EtOH extract and the EtOAc extract of the aerial parts displayed significant cytotoxicity against this cell line (IC_50_ 31.36 and 29.22 μg/mL respectively). EtOAc and EtOH extracts from the leaves, stem, and aerial parts recorded the best cytotoxic effect against HCT‐116 (IC_50_ 27.1–33.53 μg/mL) with the best effect obtained from the EtOH extract of the leaves. Extracts were less toxic to the HELA cells with the best effect exerted by the EtOAc (IC_50_ 40.91 μg/mL) and EtOH (IC_50_ 63.51 μg/mL) extracts of the leaves. Also, the same extracts, in addition to those of the aerial parts and the EtOH extract of the stem, showed the best cytotoxic effect against MDA‐MB‐231 cells with the lowest IC_50_ value (39.37 μg/mL) obtained from the EtOAc extract of the aerial parts. After detecting a significant cytotoxic effect of the ethyl acetate extract from the leaves, we conducted further experiments. First, AO/EB staining was performed using 20 μg/mL of the ethyl acetate extract. The staining results showed a reduction in cell number upon treatment with the extract (Figure 4). Additionally, Annexin V/PI staining was carried out to assess the apoptotic effect of the extract, and the results are presented in Figure 5. Moreover, cell cycle analysis demonstrated that the ethyl acetate extract induced an accumulation of cells in the subG1 phase, which is indicative of an apoptotic cell population (Figure 6). Overall, it was clear that all flower extracts almost had a weak cytotoxic effect, while the EtOAc and EtOH extracts of the other three organs revealed significant cytotoxic effects against at least one cancer cell. However, the EtOAc extract in all organs had the least phenolic content, suggesting that other non‐phenolic compounds might contribute to the cytotoxic activity. At the same time, extracts of flowers, which were less toxic, had nearly similar chemical profiles to stem and leaf extracts, and the variation was mainly quantitative, suggesting that flower extracts may not contain the appropriate amount of active molecules with cytotoxic effects or the presence of other compounds that mask the active ones. Nevertheless, some of the identified compounds were shown to exert antiproliferative effects against cancer cells. Earlier studies showed that kaempferol‐3‐glucoside inhibited the HCT‐116 cells (IC_50_ 121.845 μg/mL) (Yang et al. 2021) and MDA‐MB‐231 cells (IC_50_ 84.28 μg/mL) (Araujo‐Padilla et al. 2022). Caffeic acid inhibited the MDA‐MB‐231 (IC_50_ 150.94 μM) (Kabała‐Dzik et al. 2017). Ferulic acid suppressed the MDA‐MB‐231 cells in a concentration‐dependent manner (Zhang et al. 2016). Also, delphindin was reported to induce an anticancer effect against the HCT‐116 (Zhang et al. 2021).

**TABLE 10:** Cytotoxic effects (IC_50_ μg/mL) of *Zoegea leptaurea* extracts.

Parts	Solvents	HCT‐116	A549	HELA	MDA‐MB‐231
Flowers	EtOAc	95.80	92.64	147.73	121.52
	EtOH	118.79	121.25	105.80	132.74
	70% EtOH	356.12	298.47	321.41	288.91
	Water	248.52	285.47	262.35	301.52
Stem	EtOAc	33.53	88.94	105.80	68.96
	EtOH	30.27	96.90	134.82	59.41
	70% EtOH	198.54	241.65	256.65	178.78
	Water	325.14	332.65	345.12	313.23
Leaves	EtOAc	32.07	18.39	40.91	41.12
	EtOH	27.15	31.36	63.51	40.125
	70% EtOH	83.62	96.54	88.95	78.87
	Water	286.47	275.14	246.45	312.45
Aerial parts	EtOAc	30.41	29.22	87.01	39.37
	EtOH	31.23	86.41	103.27	52.45
	70% EtOH	189.89	178.23	201.23	212.33
	Water	205.65	245.65	232.65	237.74

### 3.6 Antibiofilm Activity

The test of minimal inhibitory concentration allowed us to identify the doses of extracts to be used in the antibiofilm test. For all extracts, the doses ranged between 35 and 80 mg/mL. Based on such results, we decided to use 20 mg/mL.

The extracts obtained from flowers and stems of *Z. leptaurea* demonstrated an excellent ability to inhibit the adhesion process of pathogenic cells (Table 11). This capability was observed against all tested pathogenic bacteria, with inhibition percentages reaching as high as 80.78% against *Acinetobacter baumannii* when flower extracts were obtained in water. Only in one case, when we tested the ethanol flower extract against *Listeria monocytogenes* , was the antibiofilm efficacy zero. It is also worth noting that, while the aqueous flower extract showed remarkable efficacy against *A. baumannii* , it exhibited low efficacy against *Klebsiella pneumoniae* . *A. baumannii* proved to be the most sensitive microbial strain to the action of all flower and stem extracts, whose inhibitory activity was never lower than 41%. *Escherichia* *coli* was only slightly less sensitive to the flower and stem extracts, with an inhibition efficacy not lower than 21.45% and reaching up to 69.77% when tested with the ethanol‐water flower extract. Regarding *L. monocytogenes* , except for the previously mentioned ethanol flower extract, the other extracts were quite effective in inhibiting the bacterial immature biofilm, with inhibition percentages reaching up to 63.42% and never falling below 34.79%. *P. aeruginosa* and *Staphylococcus aureus* were also sensitive to the extracts. In both cases, the most effective extract was the flower ethyl acetate (60.43% and 52.02%, respectively), while the least effective was the ethanol stem extract (28.52%). The significant inhibitory action exhibited on the immature biofilm did not correspond to a concomitant inhibitory activity on the mature biofilm. However, it is noteworthy that the ethyl acetate stem extract even showed an increase in inhibitory efficacy, rising from 47.77% to 63.96% against *L. monocytogenes* and from 42.17% to 79.07% against *P. aeruginosa* . *S. aureus* remained sensitive to all the extracts obtained from stems, while it is worth noting that only in two cases ( *A. baumannii* at 8.70% inhibition with flower ethanol‐water extract and *S. aureus* at 19.08% inhibition with flower ethyl acetate extract) did the extracts show an, even if lower, efficacy. *E. coli* was insensitive to extracts obtained from both flowers and stems. *K. pneumoniae* was sensitive only to the stem water extract (inhibition = 26.34%).

**TABLE 11:** Biofilm inhibitory activity (expressed as percentage) of the flower and stems extracts of *Zoegea laptaurea* added at zero (panel a CV0) and after 24 h (panel b CV24), and inhibitory activity (expressed as percentage) of flower and stems extracts added at zero (panel c MTT0) and after 24 h (panel d MTT24), of *Z. leptaurea* against the metabolism of sessile cells of the pathogens *Acinetobacter baumannii* (AB), *Escherichia* *coli* (EC), *Klebsiella pneumoniae* (KP), *Listeria monocytogenes* (LM), *Pseudomonas aeruginosa* (PA), and *Staphylococcus aureus* (SA), assuming for the control (untreated bacteria) an inhibitory value = 0).

	Flower Ethyl acetate	Flower ethanol	Flower ethan/water	Flower water	Stems ethanol	Stems eth/wat	Stems water	Stems ethyl acetate
**(a) CV0**								
AB	63.95 (±2.01)^c^	54.65 (±2.25)^b^	41.07 (±3.98)^b^	80.78 (±2.12)^c^	58.92 (±4.78)^b^	43.88 (±3.57)^b^	60.28 (±1.67)^c^	56.69 (±4.54)^b^
EC	21.45 (±2.05)^a^	50.70 (±2.35)^b^	69.77 (±2.13)^c^	52.74 (±4.54)^b^	48.95 (±3.67)^b^	33.70 (±2.22)^b^	42.35 (±2.55)^b^	38.16 (±3.08)^b^
KP	45.41 (±4.12)^b^	21.18 (±1.97)^a^	14.32 (±1.21)^a^	10.25 (±0.67)^a^	28.59 (±2.11)^b^	17.79 (±1.32)^a^	18.17 (±1.36)^a^	10.09 (±0.84)^a^
LM	56.97 (±4.31)^b^	0.00 (±0.00)^nd^	48.60 (±4.02)^b^	63.42 (±5.39)^c^	49.89 (±4.13)^b^	36.03 (±3.04)^b^	34.79 (±3.12)^b^	47.77 (±4.54)^b^
PA	60.43 (±5.56)^c^	37.27 (±2.88)^b^	48.05 (±2.54)^b^	45.73 (±2.17)^b^	27.33 (±1.98)^b^	42.02 (±3.37)^b^	44.32 (±2.76)^b^	42.17 (±3.43)^b^
SA	52.02 (±1.54)^b^	59.05 (±4.03)^b^	44.62 (±3.47)^b^	30.67 (±2.45)^b^	28.52 (±2.67)^b^	45.85 (±3.08)^b^	49.99 (±1.67)^b^	48.28 (±3.13)^b^
**(b) CV24**								
AB	0.00 (±0.00)^nd^	0.00 (±0.00)^nd^	8.70 (±0.92)^a^	0.00 (±0.00)^nd^	17.32 (±1.13)^a^	0.00 (±0.00)^nd^	11.78 (±1.67)^a^	5.10 (±0.57)^a^
EC	0.00 (±0.00)^nd^	0.00 (±0.00)^nd^	0.00 (±0.00)^nd^	0.00 (±0.00)^nd^	0.00 (±0.00)^nd^	0.00 (±0.00)^nd^	0.00 (±0.00)^nd^	0.00 (±0.00)^nd^
KP	0.00 (±0.00)^nd^	0.00 (±0.00)^nd^	0.00 (±0.00)^nd^	0.00 (±0.00)^nd^	0.00 (±0.00)^nd^	0.00 (±0.00)^nd^	26.34 (±1.13)^b^	0.00 (±0.00)^nd^
LM	0.00 (±0.00)^nd^	0.00 (±0.00)^nd^	0.00 (±0.00)^nd^	0.00 (±0.00)^nd^	0.00 (±0.00)^nd^	0.00 (±0.00)^nd^	0.00 (±0.00)^nd^	63.96 (±5.47)^c^
PA	0.00 (±0.00)^nd^	0.00 (±0.00)^nd^	0.00 (±0.00)^nd^	0.00 (±0.00)^nd^	0.00 (±0.00)^nd^	6.32 (±0.13)^a^	0.00 (±0.00)^nd^	79.07 (±6.54)^c^
SA	19.08 (±1.57)^a^	0.00 (±0.00)^nd^	0.00 (±0.00)^nd^	0.00 (±0.00)^nd^	10.18 (±0.44)^a^	19.42 (±2.04)^a^	19.44 (±1.76)^a^	7.66 (±0.57)^a^
**(c) MTT0**								
AB	10.48 (±0.95)^a^	58.20 (±4.37)^c^	55.62 (±4.89)^b^	86.57 (±2.33)^d^	20.78 (±1.01)^a^	50.83 (±4.44)^b^	51.85 (±2.09)^b^	57.77 (±3.90)^c^
EC	0.00 (±0.00)^nd^	0.00 (±0.00)^nd^	0.00 (±0.00)^nd^	2.83 (±0.08)^a^	0.00 (±0.00)^nd^	0.00 (±0.00)^nd^	7.01 (±0.55)^a^	41.76 (±1.57)^b^
KP	0.00 (±0.00)^nd^	0.00 (±0.00)^nd^	0.00 (±0.00)^nd^	0.00 (±0.00)^nd^	1.54 (±0.02)^nd^	0.00 (±0.00)^nd^	0.00 (±0.00)^nd^	0.00 (±0.00)^nd^
LM	1.20 (±0.07)^nd^	0.00 (±0.00)^nd^	0.00 (±0.00)^nd^	0.00 (±0.00)^nd^	0.00 (±0.00)^nd^	0.00 (±0.00)^nd^	3.37 (±0.04)^a^	9.10 (±0.00)^a^
PA	14.33 (±0.45)^a^	0.00 (±0.00)^nd^	0.00 (±0.00)^nd^	0.00 (±0.00)^nd^	0.00 (±0.00)^nd^	0.00 (±0.00)^nd^	0.00 (±0.00)^nd^	36.13 (±1.78)^b^
SA	0.00 (±0.00)^nd^	22.83 (±0.00)^a^	36.38 (±0.00)^b^	7.71 (±0.00)^a^	47.25 (±0.00)^b^	3.89 (±0.00)^a^	21.59 (±0.00)^a^	47.90 (±0.00)^b^
**(d) MTT24**								
AB	7.77 (±0.54)^a^	0.00 (±0.00)^nd^	8.78 (±0.67)^a^	0.00 (±0.00)^nd^	5.96 (±0.67)^a^	0.00 (±0.00)^nd^	0.00 (±0.00)^nd^	12.91 (±0.87)^a^
EC	0.00 (±0.00)^nd^	0.00 (±0.00)^nd^	0.00 (±0.00)^nd^	0.00 (±0.00)^nd^	0.00 (±0.00)^nd^	0.00 (±0.00)^nd^	0.00 (±0.00)^nd^	0.00 (±0.00)^nd^
KP	0.00 (±0.00)^nd^	0.00 (±0.00)^nd^	0.00 (±0.00)^nd^	0.00 (±0.00)^nd^	0.00 (±0.00)^nd^	0.00 (±0.00)^nd^	4.86 (±0.34)^a^	24.28 (±1.13)^a^
LM	0.00 (±0.00)^nd^	0.00 (±0.00)^nd^	0.00 (±0.00)^nd^	0.00 (±0.00)^nd^	0.00 (±0.00)^nd^	0.00 (±0.00)^nd^	0.00 (±0.00)^nd^	0.00 (±0.00)^nd^
PA	0.00 (±0.00)^nd^	0.00 (±0.00)^nd^	0.00 (±0.00)^nd^	0.00 (±0.00)^nd^	0.00 (±0.00)^nd^	23.40 (±1.23)^a^	0.00 (±0.00)^nd^	40.36 (±3.45)^b^
SA	0.00 (±0.00)^nd^	0.00 (±0.00)^nd^	0.00 (±0.00)^nd^	0.00 (±0.00)^nd^	7.18 (±0.96)^a^	12.56 (±0.99)^a^	3.84 (±0.12)^a^	30.13 (±2.02)^b^

*Note:* Results are reported as the mean ± SD of three experiments; a—*p* < 0.05; b—*p* < 0.01; c—*p* < 0.0005 according to two‐way ANOVA.

Abbreviation: nd, not detectable.

From a metabolic perspective (Table 11), *A. baumannii* and *S. aureus* were the only microorganisms sensitive to the action of all the extracts obtained from flowers or stems. The metabolism of sessile *A. baumannii* cells, in particular, was highly sensitive to the action of the flower water extract (86.57%). Except for a mild inhibitory effect shown by flower ethyl acetate (10.48%) and ethanol stems (20.87%), the inhibitory efficacy of flower and stem extracts was always above 50%. In the case of *S. aureus* , except for the nihil effect caused by the flower ethyl acetate extract, in general, the flower and stem extracts were active although they did not act with the same efficacy; therefore, the metabolism of its sessile cells was inhibited with an inhibitory strength ranging from 22.83% to 47.90%. The fact that the aqueous flower extract exhibited an inhibitory strength against *A. baumannii* similar to the value observed in the crystal violet test suggests that its inhibitory action is almost exclusively due to its ability to suppress bacterial cell metabolism. A similar consideration could be made when evaluating the inhibition percentages recorded for the ethyl acetate stem extract in the crystal violet test (48.28% and 47.90%). In the test performed on the mature biofilm, the extracts were only mildly effective, and not always, in inhibiting the metabolism of sessile cells. Only in two cases, in the test conducted against *P. aeruginosa* and *S. aureus* using the ethyl acetate stem extract, the inhibition percentage (40.36% and 30.13%, respectively) was similar to that observed in the MTT test performed on immature biofilm. This suggests that the ethyl acetate stem extract retained its inhibitory potential even on the mature biofilm.

The crystal violet test performed on the extracts obtained from the leaves revealed that their effectiveness in inhibiting the adhesion process was lower than that of the extracts obtained from flowers and stems (Table 11). However, it is important to highlight that all leaf extracts were effective in inhibiting the immature biofilm of *L. monocytogenes* (with inhibition percentages ranging from 16.52% to 36.90%), *S. aureus* (except for the water extract, and with the others showing inhibition percentages between 13.15% and 53.55%) and especially *P. aeruginosa* , against which the inhibitory effectiveness of the extracts was never lower than 40.76%, reaching up to 71.17%. *A. baumannii* was sensitive only to the ethyl acetate extract (inhibition = 25.33%) and the aqueous extract (inhibition = 72.93%). *K. pneumoniae* was sensitive to the ethanol‐water extract (inhibition = 6.18%) and, especially, to the ethyl‐acetate extract (inhibition = 50.17%). *E. coli* , on the other hand, was always resistant to all extracts. The extracts obtained from the aerial parts of the plant were also effective against *L. monocytogenes* , acting against it (with inhibition percentages ranging from 6.02% to 36.90%), and against *P. aeruginosa* (for which they showed inhibitory effectiveness between 27.64% and 48.98%). *A. baumannii* was sensitive to the extract, albeit very weakly (inhibition = 2.70%). However, the ethanol‐water extract was highly effective (inhibition = 62.37%) in acting against the formation of this bacterium's biofilm. The MTT test, performed to evaluate the role of the extracts on the metabolism of sessile cells within the immature biofilm of *A. baumannii* , allows for some considerations: all four leaf extracts were able to interfere by inhibiting those metabolic processes that can generally transform bacterial cells, making them more virulent. This is evidenced by the fact that inhibition percentages never fell below 70% (the inhibition percentage recorded by the leaves extract obtained with ethanol), and even reached 98.76% (when we tested the aqueous leaves extract). The same can be said, albeit to a lesser extent, for three out of four extracts obtained from the aerial parts mixture, which recorded inhibition percentages no lower than 54% (ethanol/water extract), reaching inhibition values of 67.28% (in the case of the test conducted with the ethyl acetate) (Table 12). The extracts obtained from the aerial parts of the plant were less effective, not higher than 67.28% (ethyl acetate extract). Comparing the results with the crystal violet test conducted on the immature biofilm, we can hypothesize that, in most cases, even if the extracts could do little or nothing against the biofilm, they were at least extremely effective in affecting bacterial cell metabolism. The data also indicate that the ethyl acetate extract, but especially the aqueous leaf extract, acted both on the biofilm and on the metabolism of *A. baumannii* sessile cells. A similar reasoning can be made for *E. coli* : while it was completely insensitive to the action of leaf and aerial part extracts on its immature biofilm, it was sensitive when its sessile cell metabolism was evaluated in the MTT test. The data of the MTT test suggest that, with the exception of the aqueous leaf extract, the other extracts, while failing to block the biofilm formation process of *E. coli* , at least managed to limit the metabolic events occurring within its sessile cells. The aqueous leaf extract, however, proved to be completely effective both in blocking the immature biofilm and in acting against the metabolism of its sessile cells. The extracts failed to inhibit the metabolic changes occurring in the sessile cells of *K. pneumoniae* , meaning they were almost always ineffective against it both in terms of biofilm inhibition (except for the ethyl acetate extract, which recorded an inhibitory percentage of 50.97%, and the ethanol/water extracts, which inhibited the immature biofilm with a percentage of 6.18%) and metabolic activity. *L. monocytogenes* sessile cells were particularly sensitive to the ethyl acetate and ethanol flower extracts, with inhibition percentages of 45.19% and 44.70%, respectively, as well as to the ethyl acetate extract and the ethanol‐water extract of the aerial parts, with inhibition percentages of 24.54% and 29.39%, respectively. Thus, in most cases, the extracts were mainly able to act on the metabolism of sessile cells within the immature biofilm, despite a few exceptions in biofilm inhibition. Apart from a few exceptions, the flower extracts and the aerial parts mixture extracts were effective in acting against the metabolism of *P. aeruginosa* sessile cells, with inhibition percentages reaching 41.93% (ethyl acetate flower extract) and 42.50% (ethyl acetate extract of the aerial parts) (Table 12). This suggests that the action of these extracts, which already exhibited good biofilm inhibitory activity, is primarily exerted through the blocking or significant limitation of metabolic changes within sessile cells. The results of the crystal violet and MTT tests on *S. aureus* immature biofilm indicate that the inhibitory action of leaf and aerial parts mixture extracts could be partially attributable to their effect on cell metabolism, being in some cases completely ineffective. Thus, where the crystal violet test yielded positive results, the action can be generally attributed to other biofilm‐inhibiting mechanisms.

**TABLE 12:** Biofilm inhibitory activity (expressed as percentage) of the leaves and aerial part extracts of *Zoegea laptaurea* added at zero (panel a CV0) and after 24 h (panel b CV24), and inhibitory activity (expressed as percentage) of flower and stem extracts added at zero (panel c MTT0) and after 24 h (panel d MTT24), of *Z. leptaurea* against the metabolism of sessile cells of the pathogens *Acinetobacter baumannii* (AB), *Escherichia* *coli* (EC), *Klebsiella pneumoniae* (KP), *Listeria monocytogenes* (LM), *Pseudomonas aeruginosa* (PA), and *Staphylococcus aureus* (SA), assuming for the control (untreated bacteria) an inhibitory value = 0.

	Leaves‐Ethyl acetate	Leaves‐Ethanol‐	Leaves‐Eth/Wat	Leaves‐wate	Leaves‐wate	Aerial parts (mix)‐ Ethanol	Aerial parts (mix)‐ Ethanol/Water	Aerial parts (mix)‐ Water
**(a) CV0**								
AB	25.33 (±2.04)^b^	0.00 (±0.00)^nd^	0.00 (±0.00)^nd^	72.93 (±4.44)^c^	0.00 (±0.00)^nd^	0.00 (±0.00)^nd^	2.70 (±0.15)^a^	0.00 (±0.00)^nd^
EC	0.00 (±0.00)^nd^	0.00 (±0.00)^nd^	0.00 (±0.00)^nd^	0.00 (±0.00)^nd^	0.00 (±0.00)^nd^	0.00 (±0.00)^nd^	0.00 (±0.00)^nd^	0.00 (±0.00)^nd^
KP	50.97 (±4.09)^b^	0.00 (±0.00)^nd^	6.18 (±0.12)^a^	0.00 (±0.00)^nd^	0.00 (±0.00)^nd^	0.00 (±0.00)^nd^	0.00 (±0.00)^nd^	0.00 (±0.00)^nd^
LM	16.52 (±0.85)^a^	31.11 (±2.08)^b^	36.90 (±3.44)^b^	26.99 (±2.06)^b^	32.87 (±2.71)^b^	18.00 (±0.88)^a^	6.02 (±0.23)^a^	6.67 (±0.14)^a^
PA	71.17 (±4.65)^c^	51.33 (±4.32)^b^	44.30 (±3.77)^b^	40.76 (±3.90)^b^	27.64 (±2.01)^b^	41.76 (±3.14)^b^	46.48 (±3.21)^b^	48.98 (±3.76)^b^
SA	16.53 (±1.02)^a^	53.55 (±4.45)^b^	13.15 (±1.05)^a^	0.00 (±0.00)^nd^	6.45 (±0.24)^a^	7.54 (±0.65)^a^	62.37 (±4.32)^c^	0.00 (±0.00)^nd^
**(b) CV24**								
AB	6.27 (±0.51)^a^	0.00 (±0.00)^nd^	33.24 (±2.03)	6.12 (±0.32)^a^	0.89 (±0.42)^nd^	19.27 (±1.14)^a^	20.10 (±1.98)^a^	0.00 (±0.00)^nd^
EC	0.00 (±0.00) ^nd^	0.00 (±0.00)^nd^	40.55 (±3.35)^b^	0.00 (±0.00)^nd^	9.84 (±0.19)^a^	29.01 (±2.05)^b^	10.23 (±0.11)^a^	0.00 (±0.00)^nd^
KP	32.30 (±2.65)^b^	0.18 (±0.03)^nd^	15.47 (±1.05)^a^	0.00 (±0.00)	7.89 (±0.43)^a^	16.89 (±0.87)^a^	20.87 (±1.75)^a^	14.91 (±0.19)^a^
LM	0.00 (±0.00)^nd^	0.00 (±0.00)^nd^	12.68 (±0.87)^a^	16.53 (±0.45)^a^	24.02 (±2.01)^a^	0.00 (±0.00)^nd^	35.88 (±3.22)^b^	28.83 (±2.45)^b^
PA	11.72 (±0.65)^a^	6.92 (±0.61)^a^	0.28 (±0.03)^nd^	0.00 (±0.00)^nd^	59.67 (±4.87)^c^	63.96 (±4.01)^c^	49.06 (±4.09)^b^	12.81 (±0.96)^a^
SA	41.06 (±3.01)^b^	20.56 (±1.75)^a^	56.03 (±2.25)^c^	55.07 (±3.91)^c^	45.14 (±3.45)^b^	42.43 (±3.04)^b^	63.46 (±3.11)^c^	57.27 (±3.66)^c^
**(c) MTT0**								
AB	73.16 (±2.67)^c^	70.02 (±3.33)^c^	78.92 (±2.98)^c^	98.76 (±0.02)^d^	67.28 (±3.65)^c^	59.71 (±3.34)^c^	54.42 (±4.43)^b^	14.25 (±0.67)^a^
EC	36.62 (±0.57)^b^	24.68 (±1.23)^a^	27.23 (±1.17)^a^	0.00 (±0.00)^nd^	31.46 (±2.09)^b^	44.44 (±3.03)^b^	16.69 (±0.56)^a^	8.89 (±0.07)^a^
KP	0.00 (±0.00)^nd^	0.00 (±0.00)^nd^	0.00 (±0.00)^nd^	0.00 (±0.00)^nd^	0.00 (±0.00)^nd^	0.00 (±0.00)^nd^	0.00 (±0.00)^nd^	0.00 (±0.00)^nd^
LM	45.19 (±0.00)^b^	44.70 (±0.00)^b^	8.02 (±0.00)^a^	21.17 (±0.00)^a^	24.54 (±0.00)^a^	19.38 (±0.00)^a^	29.39 (±0.00)^b^	9.76 (±0.00)^a^
PA	41.93 (±0.00)^b^	39.45 (±0.00)^b^	2.12 (±0.00)^a^	0.00 (±0.00)	42.50 (±0.00)^b^	0.00 (±0.00)^nd^	0.00 (±0.00)^nd^	17.15 (±0.00)^a^
SA	0.00 (±0.00)^nd^	33.16 (±1.43)^b^	0.00 (±0.00)^nd^	0.00 (±0.00)^nd^	32.60 (±2.21)^b^	20.80 (±1.76)^a^	7.71 (±0.12)^a^	18.35 (±0.45)^a^
**(d) MTT24**								
AB	83.40 (±2.11)^d^	50.97 (±4.01)^b^	33.02 (±2.03)^b^	21.21 (±1.42)^a^	35.65 (±2.24)^b^	21.95 (±1.04)^a^	26.11 (±2.06)^b^	16.56 (±0.34)^a^
EC	62.72 (±3.98)^c^	44.06 (±3.01)^b^	45.29 (±3.34)^b^	1.53 (±0.02)^nd^	50.64 (±4.22)^b^	44.91 (±3.62)^b^	34.32 (±3.04)^b^	0.10 (±0.03)^nd^
KP	79.47 (±3.76)^c^	54.74 (±4.33)^b^	36.94 (±1.57)^b^	1.60 (±0.04)^nd^	49.28 (±1.67)^b^	36.59 (±1.13)^b^	43.93 (±3.65)^b^	19.52 (±0.92)^a^
LM	62.07 (±2.44)^c^	40.06 (±3.02)^b^	29.29 (±0.67)^b^	56.38 (±1.13)^c^	0.00 (±0.00)^nd^	44.81 (±3.09)^b^	24.24 (±2.01)^a^	28.91 (±1.17)^b^
PA	37.41 (±3.02)^b^	48.73 (±4.02)^b^	46.54 (±1.67)^b^	25.13 (±1.18)^a^	0.00 (±0.00)^nd^	0.00 (±0.00)^nd^	0.00 (±0.00)^nd^	2.21 (±0.06)^a^
SA	49.79 (±3.22)^b^	10.81 (±0.34)^a^	10.96 (±0.55)^a^	0.00 (±0.00)^nd^	20.71 (±1.02)^a^	0.00 (±0.00)^nd^	37.87 (±2.09)^b^	0.00 (±0.00)^nd^

*Note:* Results are reported as the mean ± SD of three experiments; a—*p* < 0.05; b—*p* < 0.01; c—*p* < 0.0005 according to two‐way ANOVA.

Abbreviation: nd, not detectable.

It is very interesting to highlight the behavior exhibited by the extracts from leaves and aerial parts in the crystal violet test conducted on the mature biofilm, that is, when they were added to bacterial cultures after 24 h of bacterial growth, which implies a well‐established biofilm (Table 12). Indeed, for example, the mature biofilm of *E. coli* , which during the adhesion phase was completely resistant to the presence of extracts obtained from flowers and aerial parts, exhibited a certain sensitivity to the presence of the same extracts, with inhibition percentages ranging from 9.84% to 40.55%. The same considerations can be made for the mature biofilm of *A. baumannii* , against which the extracts showed an inhibitory performance reaching 33.24%. In the case of *K. pneumoniae* , whose immature biofilm was almost always resistant to the extracts, we observed greater sensitivity when the test was conducted on the mature biofilm, with an inhibition percentage reaching 32.30%. The mature biofilm of *S. aureus* demonstrated greater sensitivity to the extracts compared to the immature biofilm, as evidenced by the fact that, in the test conducted with the leaves extracts, the inhibition percentage never dropped below 20.56%, reaching up to 56.03%. The extracts obtained from the aerial parts were able to effectively inhibit the mature biofilm of *S. aureus* , with inhibition percentages never below 42.43% and reaching up to 63.96%. *S. aureus* , whose immature biofilm was resistant to the aqueous extracts of flowers and aerial parts, showed on the contrary sensitivity to the same extracts when the test was conducted on the mature biofilm, with the inhibitory action reaching 55.07% (aqueous flower extract) and 57.27% (aqueous extract of the aerial parts). This also suggests that the extraction method in this case favored greater inhibitory performance against this bacterium. The MTT test on mature biofilm strongly suggests a vigorous inhibitory effect exerted by leaves extracts on biofilm metabolism, with a significant percentage of metabolic inhibition. Ethyl acetate extracts were particularly effective, with inhibition never falling below 37.41% (against *P. aeruginosa* ) and reaching up to 83.40% (against *A. baumannii* mature biofilm). Leaves extracts generally outperformed those from the aerial parts mixture, especially against *S. aureus* and *K. pneumoniae* . Given the weak action exhibited by the extracts in general on the mature biofilm of almost all the tested microbial strains, as resulting from the crystal violet test, the high percentages of inhibition arising from the MTT test let us state, once again, that, in many cases, the inhibitory activity of the extracts was mainly expressed through their ability to block the metabolism of the mature bacterial biofilm. This condition, by inducing specific metabolic changes in the cells, leads to increased virulence. Along with the persistence of a mature biofilm, this represents a high risk of irreversibility, making it more difficult for conventional medical treatments to eradicate the infection.

### 3.7 Effect of the Different Extracts on Probiotic Growth

In addition to assessing antibiofilm activity, we also investigated whether the extracts, when added to MRS broth, could influence the growth of five probiotic strains. These strains are either commercially available or, as in the case of *Lactobacillus bulgaricus* , belong to international culture collections with well‐documented probiotic properties. The results (Table 13) are expressed as a percentage, considering the bacterial growth in standard MRS broth (without extracts) as 100%. The growth response of the five probiotic strains varied depending on the type of extract incorporated into the growth medium.

**TABLE 13:** Percentage of growth of probiotics, performed in the presence of 20 μg/mL of the extracts, compared to the corresponding control grown in the conventional MRS broth (assuming for control a growth = 100%).

	Flower ethyl acetate	Flower ethanol	Flower ethan/water	Flower water	Stems ethanol	Stems eth/wat	Stems water	Stems ethyl acetate
LB	82.04 (±2.02)^a^	50.12 (±1.57)^b^	25.67 (±2.01)^b^	80.57 (±1.98)^a^	72.23 (±3.13)^a^	46.96 (±3.07)^b^	76.59 (±4.11)^a^	20.82 (±1.13)^b^
LC	83.14 (±1.44)^a^	79.18 (±1.66)^a^	64.77 (±4.04)^a^	68.65 (±4.12)^a^	120.70 (±2.44)^a^	97.93 (±2.23)^nd^	84.33 (±3.41)^a^	60.22 (±4.98)^a^
LG	105.80 (±1.32)^a^	94.69 (±1.57)^a^	92.20 (±1.17)^a^	87.88 (±3.54)^a^	117.67 (±6.07)^a^	106.04 (±3.23)^a^	89.68 (±3.09)^a^	84.57 (±4.04)^a^
LP	134.19 (±7.12)^a^	108.55 (±2.99)^a^	90.23 (±3.07)^a^	91.81 (±3.88)^a^	183.10 (±3.34)^b^	117.01 (±6.55)^a^	106.35 (±2.25)^a^	81.92 (±1.56)^a^
LR	112.93 (±1.87)^a^	106.20 (±2.09)^a^	106.81 (±2.23)^a^	103.67 (±1.57)^a^	127.97 (±8.55)^a^	119.55 (±3.14)^a^	105.12 (±1.57)^a^	106.61 (±1.54)^a^

	Leaves‐ethyl acetate	Leaves‐ethanol	Leaves‐Eth/Wat	Leaves‐water	Aerial parts (mix)‐Ethyl acetate‐	Aerial parts (mix)‐ethanol	Aerial parts (mix)‐eth/Wat	Aerial parts (mix)‐water
LB	135.44 (±1.84)^a^	132.69 (±8.43)^a^	93.47 (±4.57)^a^	100.98 (±1.12)^nd^	121.17 (±5.56)^a^	97.67 (±1.57)^a^	91.41 (±2.01)^a^	99.85 (±1.02)^a^
LC	234.63 (±10.32)^c^	106.34 (±2.27)^a^	79.79 (±1.57)^a^	54.54 (±1.13)^b^	115.08 (±5.87)^a^	64.83 (±3.02)^a^	91.43 (±1.04)^a^	87.07 (±3.32)^a^
LG	114.96 (±5.01)^a^	105.14 (±2.01)^a^	25.22 (±1.02)^b^	106.94 (±1.44)^a^	119.69 (±8.04)^a^	115.53 (±1.57)^a^	119.20 (±4.06)^a^	106.59 (±5.45)^a^
LP	279.83 (±15.08)^c^	147.36 (±8.78)^b^	126.72 (±6.64)^a^	69.85 (±5.02)^a^	149.35 (±12.24)^b^	112.30 (±5.09)^a^	73.95 (±3.13)^a^	84.89 (±3.33)^a^
LR	185.92 (±11.02)^b^	158.90 (±11.01)^b^	414.49 (±24.02)^d^	146.17 (±9.04)^b^	216.53 (±12.44)^c^	550.48 (±32.81)^d^	616.30 (±24.87)^d^	544.32 (±22.56)^d^

*Note:* Different letters (a–d) indicate significant differences in the action of the extracts (*p* ≤ 0.05).

Abbreviations: LB, *Lactobacillus bulgaricus*; LC, *Lactocaseobacillus casei* Shirota; LG, *Lactobacillus gasseri* ; LP, *Lactiplantibacillus plantarum*; LR, *Lacticaseibacillus rhamnosus*.

The analysis of probiotic growth in the presence of flower and stem extracts revealed that not all bacteria benefited from the presence of flower and stem extracts. Data comparing bacterial growth with and without extracts indicated that, among the flower extracts of *Z. leptaurea*, the ethyl acetate extract had a positive effect on *L. gasseri* , *L. plantarum* , and *L. rhamnosus* . Their growth increased by 5.80% ( *L. gasseri* ) to 34.19% compared to the control, which was set at 100%. The ethanolic flower extract also positively influenced *L. plantarum* (*D* = 8.55%) and *L. rhamnosus* (*D* = 6.20%). Additionally, *L. rhamnosus* responded positively to the aqueous flower extract of *Z. leptaurea*. These findings suggest that for *L. plantarum* and *L. rhamnosus* , the growth‐promoting effect of *Z. leptaurea* flower extracts is influenced by their polarity. The most pronounced beneficial effect was observed with the ethyl acetate extract, while the aqueous extract, although still effective, showed a weaker impact. A similar trend was observed for the other three bacterial strains; however, in these cases, the flower extracts had a negative impact on the growth of *L. bulgaricus* , *L. casei Shirota*, and *L. gasseri* (except when *L. gasseri* was grown with the ethyl acetate flower extract).

When the probiotic strains were incubated in MRS broth supplemented with stem extracts of *Z. leptaurea*, a positive effect was once again observed, particularly in *L. rhamnosus* . Its growth increased by 5.12% with the aqueous extract and by 27.97% with the ethanolic extract. This suggests that ethanol extraction facilitates the recovery of polyphenols (or a higher concentration of them) that are beneficial for *L. rhamnosus* growth. A similar effect was observed for *L. casei Shirota*, where the ethanolic extract was the only stem extract that increased growth (*D* = 20.70%), as well as for *L. gasseri* (*D* = 17.67%) compared to the control. The polyphenol composition data suggest that kaempferol‐3‐glucoside may have contributed to the extracts' ability to promote bacterial growth. This effect was particularly evident in *L. plantarum* , which exhibited an 83% increase in growth when exposed to the ethanolic stem extract compared to the control grown in conventional MRS broth. For *L. bulgaricus* , although the ethanolic stem extract did not enhance growth beyond control levels, it at least mitigated the inhibitory effect observed with other extracts.

The presence of extracts obtained from *Z. leptaurea* leaves and aerial parts led to different responses depending on the probiotic strain and the extraction method used. In the case of leaf extracts, the ethyl acetate and ethanolic extracts were particularly effective in promoting probiotic growth, with increases (*D*) ranging from 14.96% ( *L. gasseri* in the presence of the ethyl acetate extract) to 85.92% ( *L. rhamnosus* in the presence of the ethyl acetate extract), reaching as high as 134.63% (*L. casei Shirota* with the ethyl acetate extract). Notably, the leaf extract obtained using the ethanol/water mixture significantly enhanced *L. rhamnosus* growth, increasing from 100% (control) to 414.49%. This extract also favored the growth of *L. plantarum* (*D* = 26.72%). When the extracts from the aerial parts of the plant were tested, the effects were even more striking. The addition of the ethanol/water aerial part extract to *L. rhamnosus* culture medium led to an extraordinary growth increase of 616.30%. A similar effect was observed with the ethanolic extract (550.48%) and, to a lesser extent, with the aqueous extract (~544%) and the ethyl acetate extract (which still resulted in a 216% increase). Overall, the data strongly suggest that extracts derived from the leaves and aerial parts of *Z. leptaurea* have the most significant positive impact on the growth of the five probiotic strains tested.

Plants and their derivatives are a crucial source of bioactive compounds, such as polyphenols, which can help combat some of the most harmful microorganisms to human health—many of which are increasingly resistant to conventional antibiotics. Conversely, these compounds can also support the growth of bacteria of beneficial and technological interest (Colautti et al. 2024; Nazzaro et al. 2024; Pannella et al. 2020). Selecting the appropriate plant matrices, extraction techniques, and solvents is therefore essential to maximizing the yield of these important secondary metabolites. *Z. leptaurea* is a plant that, from a microbiological perspective, has been studied to a very limited extent. Jawad et al. (1988) reported a certain degree of antimicrobial activity in the ethanolic extracts of this plant against some clinically relevant bacteria and fungi. However, studies on *Z. leptaurea* have mainly focused on its antifungal properties (Nawrot et al. 2020).

In our study, we aimed to assess the microbiological effectiveness of *Z. leptaurea* by analyzing extracts obtained from different parts of the plant (flowers, leaves, stems, and a mix of aerial parts). We used four different extraction solvents: water, ethanol/water, ethanol, and ethyl acetate. To our knowledge, beyond the already limited antibacterial studies on this species, no research has been conducted to specifically evaluate the role of *Z. leptaurea* extracts or the differences in antibiofilm activity depending on the plant part and extraction method used. This confirms findings from previous studies on *Tordylium apulum* (Zengin et al. 2024) and *Leonurus cardiaca* (Nilofar et al. 2024). A correlation analysis between biofilm inhibition activity and polyphenolic composition revealed that certain flavonoids significantly influenced the antibiofilm effect of the flower extract against *E. coli* . These included isoquercitrin (*r* = 0.88), delphinidin 3,5‐diglucoside (*r* = 0.85), and kaempferol 3‐glucoside (*r* = 0.85) as well as chlorogenic and neochlorogenic acids (*r* = 0.83 and 0.80, respectively). The antibiofilm activity of flavonoids has been widely recognized, while phenolic acids have also demonstrated antibiofilm properties against *E. coli* , as reported by Bernal‐Machado et al. (2018).

The strong inhibitory effect of the stem extracts against immature *A. baumannii* biofilms may be attributed to the significant influence of nearly all the analyzed polyphenols, with correlation values ranging from 0.80 (rutin) to 0.98 (ferulic acid and syringic acid). The relatively weak inhibition observed against mature *A. baumannii* biofilms was largely influenced by flavonoids such as isoquercitrin (*r* = 0.92), delphinidin 3,5‐diglucoside (*r* = 0.90), and kaempferol 3‐glucoside (*r* = 0.89). The role of rutin, isoquercitrin, and certain phenolic acids aligns with findings by Ivanov et al. (2022), who confirmed their significant antibiofilm potential. These compounds could, therefore, serve as promising future agents in the fight against the increasing virulence of pathogenic bacteria (Bhagwat et al. 2022). Additionally, isoquercitrin and delphinidin 3,5‐diglucoside exhibited strong or excellent enhancement of the inhibitory effect of the leaf extract against immature *L. monocytogenes* biofilms (*r* = 0.95). Though less effective, they also contributed to inhibiting mature *A. baumannii* biofilms (*r* = 0.80). However, isoquercitrin did not seem to influence the extracts' ability to inhibit sessile cell metabolism, as its correlation values were mostly negative. This suggests that isoquercitrin acts through mechanisms other than metabolic interference, possibly structural or genetic. Phenolic acids, including neochlorogenic acid, chlorogenic acid, and 4‐hydroxybenzoic acid, exhibited high correlation values (*r* = 0.80, 0.83, and 0.75, respectively) with the inhibitory activity of *Z. leptaurea* flower extracts against immature *E. coli* biofilms. The fact that an even stronger correlation was observed in relation to the inhibition of *E. coli* sessile cell metabolism suggests that these compounds, particularly chlorogenic acid, may play a role in biofilm inhibition through multiple mechanisms (Monte et al. 2014), including metabolic disruption. Similarly, although neochlorogenic acid has been studied less extensively, it has demonstrated antibiofilm activity against several pathogens, including *Stenotrophomonas maltophilia* (Zhang et al. 2019), primarily by affecting the bacterial cell membrane. Our correlation analysis suggests, for the first time, that neochlorogenic acid may also influence the metabolic pathways of sessile cells, potentially preventing or limiting their ability to become more virulent.

The positive effect of most of the extracts of *Z. leptaurea* to the five probiotics considered as tester strains was studied herein for the first time; thus, we do not have any other research to compare the effect. However, considering the polyphenol composition of the extracts, in the results section, we highlighted the influence of kaempferol 3 glucoside on the positive effect exhibited by some extracts on probiotic growth. The presence of a significant amount of such a metabolite can be significant. Thus, it can stimulate the growth of some beneficial bacteria, such as *L. plantarum* , and probably affect the production of postbiotic metabolites, which can exhibit healthy properties. Future works will be aimed at studying the impact that the fermentation of these extracts can have on their phenolic composition and some biological properties, such as their antimicrobial and antioxidant activity. Generally, phenolic compounds from various classes are transformed into derivatives with greater bioactivity than their original forms. In different studies, such as that of Leonard et al. (2021), fermentation has been shown to enhance phenolic content and boost antioxidant activity. Therefore, De Montijo‐Prieto et al. (2023) demonstrated a positive effect of the fermentation of the avocado leaf extract by lactic acid bacteria, including *L. plantarum*. Our future study could constitute the basis for future application in the development of new functional foods or ingredients (Coelho et al. 2024).

## 4 Conclusions

It is worth mentioning that this is the first study to investigate the phenolic content and biological activities of a member of the genus *Zoegea*. Results indicated that *Z. leptaurea* was rich in phenolics and possessed significant antioxidant, enzyme inhibitory, and cytotoxic activity. The observed differences in biological activities among the extracts are mainly linked to the extraction solvent employed. Generally, the antioxidant activity was positively correlated to their phenolics, where 70% of EtOH recovered the highest content. The highest enzyme inhibitory and anticancer activities were observed in EtOAc and EtOH extracts, suggesting that other non‐phenolic compounds might also participate in these activities. In addition, the extracts showed significant anti‐biofilm and probiotic effects. Thus, these findings provided helpful information on the potential of *Z. leptaurea* as a promising source of bioactive compounds with a broad range of therapeutic benefits. The isolation of bioactive molecules responsible for the observed biological activities, including non‐phenolic compounds, and understanding their mechanism of action (bioavailability, etc.) are recommended. Also, further research is needed to understand the extent of its medicinal benefits and possible applications in the food, cosmetic, and pharmaceutical industries.

## Author Contributions

**Sakina Yagi:** methodology, investigation, data curation, visualization, writing – original draft, writing – review and editing, and investigation. **Giovanni Caprioli:** investigation, data curation, writing – original draft, and writing – review and editing. **Gabriele Rocchetti:** data curation, visualization, writing – original draft, and writing – review and editing. **Filomena Nazzaro:** methodology, investigation, data analysis, writing – original draft, writing – review and editing, and investigation. **Florinda Fratianni:** methodology, investigation, data analysis, writing – original draft, writing – review and editing, and investigation. **Francesca Coppola:** methodology, investigation, data analysis, writing – original draft, writing – review and editing, and investigation. **Ozgur Yuksekdag:** methodology, investigation, data curation, visualization, writing – original draft, and investigation. **Ismail Koyuncu:** methodology, investigation, data curation, visualization, writing – original draft, and investigation. **Laura Acquaticci:** investigation, data curation, writing – original draft, writing – review and editing. **Simone Angeloni:** investigation, data curation, writing – original draft, writing – review and editing. **Mehmet Maruf Balos:** methodology, investigation, and resources. **Ulku Yerebasan:** methodology, investigation, and resources. **Gokhan Zengin:** methodology, investigation, data curation, writing – original draft, writing – review and editing, and investigation.

## Conflicts of Interest

The authors declare no conflicts of interest.

## Supporting information


Data S1

## Data Availability

Data will be made available on request.
