# Supplementary material for: Opening New Roads for Multi‐Directional Functional Applications Through In Vitro Chemical and Biological Analysis of Zoegea leptaurea L. Extracts
Source: Food Sci Nutr. 2025 May 13;13(5):e70261. doi: 10.1002/fsn3.70261 (PMC12069977; doi:10.1002/fsn3.70261)
Supplement: Supplementary file 1 — Data S1 [file FSN3-13-e70261-s001.docx]

**LC-MS-qTOF metabolomic analysis**

Metabolomic studies were performed using an Agilent 1290 Infinity II liquid chromatography (LC) system coupled to an Agilent 6546 LC/MS QTOF mass spectrometer (Agilent, USA). Metabolite separation was performed using a Poroshell 120 EC-C18 column (2 × 150 mm, 2.7 µm, Agilent, USA). The mobile phase consisted of 0.1% formic acid in water (solvent A) and methanol, using a gradient elution approach in which the solvent composition varied over time as follows: 0-4 min, 85% A; 4-7 min, 75% A; 7-9 min, 68% A; 9-16 min, 60% A; 16-22 min, 45% A; 22-28 min, 5% A; and 28-30 min, 5% A. The flow rate was maintained at 0.5 mL/min while the column temperature was set at 35°C. A sample volume of 1 µL was injected into the system for each analysis. The mass spectrometry system was operated in both positive and negative ionization modes, with the QTOF instrument parameters optimized as follows: a scan range of 100-1000 m/z, a drying gas temperature of 160°C, and a sheath gas flow rate of 12.0 L/min. The capillary and nozzle voltages were set to 5.0 kV and 2.0 kV, respectively. In addition, the fragmentor voltage was set to 140 V and collision energies of 10, 20, and 40 eV were applied. MS/MS data acquisition was performed in the 50-800 m/z range using a retention time window of 0.5 min and an isolation width of 1.3 amu, with spectra collected at a rate of three per second. Two internal reference masses (112.9855 m/z and 966.0007 m/z) were continuously monitored throughout the experiment to ensure accurate mass correction. Mass Hunter Profinder 10.0 software was used for data processing to extract features and align chromatographic peaks. Chromatography alignment parameters were configured with a minimum signal intensity of 1000 counts and a maximum retention time error of 0.5 minutes. The feature extraction process was restricted to an m/z range of 100-1000, with retention time errors limited to 0.25 minutes and mass errors limited to 2 mDa.

The online platform MetaboAnalyst (https://www.metaboanalyst.ca/) was used for statistical analysis. Partial Least Squares Discriminant Analysis (PLS-DA) and Variable Importance in Projection (VIP) plots for both positive and negative ionization modes were performed to enhance the identification of key discriminating variables between different extracts. In addition, targeted MS/MS fragmentation analysis was performed to identify potential metabolites. The obtained spectral data were compared with the Metline database, internal spectral libraries and literature sources using retention time and MS/MS fragmentation patterns as reference criteria.

**Table S1**

HPLC–MS/MS acquisition parameters (dynamic-MRM mode) used for the analysis of the 38 marker compounds.

| No. | Compounds | Precursor ion, *m/z* | Product ion, *m/z* | Fragm-entor, V | Collision energy, V | Polarity | Retention time (Rt, min) | Delta retention time (ΔRt) |
| --- | --- | --- | --- | --- | --- | --- | --- | --- |
| 1 | Gallic acid | 169 | 125.2^*^ | 97 | 12 | Negative | 6.96 | 2 |
| 2 | Neochlorogenic acid | 353 | 191.2^*^, 179 | 82 | 12, 12 | Negative | 9.52 | 2 |
| 3 | Delphindin-3-galactoside | 465.01 | 303^*^ | 121 | 20 | Positive | 11.36 | 2 |
| 4 | (+)-Catechin | 289 | 245.2^*^,109.2 | 131 | 8, 20 | Negative | 11.44 | 2 |
| 5 | Procyanidin B2 | 576.99 | 576.99^*^, 321.2 | 160 | 0, 32 | Negative | 12.41 | 2 |
| 6 | Chlorogenic acid | 353 | 191.2^*^, 127.5 | 82 | 12, 20 | Negative | 12.42 | 2 |
| 7 | *p*-Hydroxybenzoic acid | 137 | 93.2^*^ | 92 | 16 | Negative | 12.86 | 2 |
| 8 | (-)-Epicatechin | 289 | 245.1^*^, 109.1 | 126 | 8, 20 | Negative | 13.03 | 2 |
| 9 | Cyanidin-3-glucoside | 449 | 287.3^*^, 255.6 | 121 | 20, 20 | Positive | 13.14 | 2 |
| 10 | Petunidin-3-glucoside | 479.01 | 317^*^, 302 | 121 | 20, 44 | Positive | 13.26 | 2 |
| 11 | 3-Hydroxybenzoic acid | 137 | 93.2^*^ | 88 | 8 | Negative | 13.59 | 2 |
| 12 | Caffeic acid | 179 | 135.2^*^, 134.1 | 92 | 12, 24 | Negative | 13.65 | 2 |
| 13 | Vanillic acid | 167 | 152.4^*^, 108.1 | 88 | 12, 20 | Negative | 14.32 | 2 |
| 14 | Resveratrol | 227 | 185^*^ | 131 | 12 | Negative | 14.40 | 2 |
| 15 | Pelargonidin-3-glucoside | 433.01 | 271^*^, 121 | 116 | 24, 50 | Positive | 14.52 | 2 |
| 16 | Pelagonidin-3-rutinoside | 579.01 | 271^*^ | 145 | 32 | Positive | 14.56 | 2 |
| 17 | Malvidin-3-galactoside | 493.01 | 331^*^, 315.1 | 121 | 20, 50 | Positive | 14.64 | 2 |
| 18 | Syringic acid | 196.9 | 182.2^*^, 121.2 | 93 | 8, 12 | Negative | 15.28 | 2 |
| 19 | Procyanidin A2 | 575 | 575^*^, 285 | 170 | 0, 20 | Negative | 16.18 | 2 |
| 20 | *p*-Coumaric acid | 163 | 119.2^*^, 93.2 | 83 | 12, 36 | Negative | 16.70 | 2 |
| 21 | Ferulic acid | 193 | 134.2^*^, 131.6 | 83 | 12, 8 | Negative | 17.10 | 2 |
| 22 | 3,5-Dicaffeoylquinic acid | 514.9 | 353.1^*^, 191 | 117 | 8, 28 | Negative | 17.61 | 2 |
| 23 | Rutin | 609 | 300.2^*^, 271.2 | 170 | 32, 50 | Negative | 17.73 | 2 |
| 24 | Hyperoside | 465.01 | 303^*^, 61.1 | 97 | 8, 50 | Positive | 18.33 | 2 |
| 25 | Isoquercitrin | 463 | 271.2^*^, 300.2 | 155 | 44, 24 | Negative | 18.36 | 2 |
| 26 | Delphindin-3,5-diglucoside | 462.9 | 300.1^*^ | 165 | 24 | Negative | 18.38 | 2 |
| 27 | Phloridzin | 435.39 | 273^*^, 167 | 155 | 8, 28 | Negative | 18.83 | 2 |
| 28 | Quercitrin | 446.99 | 300.2^*^, 301.2 | 160 | 24, 16 | Negative | 19.61 | 2 |
| 29 | Myricetin | 316.99 | 179.1^*^, 182 | 150 | 16, 24 | Negative | 19.61 | 2 |
| 30 | Naringin | 578.99 | 271.3^*^, 151.3 | 170 | 32, 44 | Negative | 19.62 | 2 |
| 31 | Kaempferol-3-glucoside | 447 | 284.2^*^, 255.2 | 170 | 24, 40 | Negative | 19.77 | 2 |
| 32 | Hesperidin | 611.01 | 303^*^, 334.8 | 112 | 20, 12 | Positive | 20.19 | 2 |
| 33 | Ellagic acid | 301 | 301^*^, 229 | 170 | 0, 24 | Negative | 21.41 | 2 |
| 34 | *trans*-cinnamic acid | 149 | 131.2 | 74 | 4 | Positive | 21.44 | 2 |
| 35 | Quercetin | 300.99 | 151.2^*^, 179.2 | 145 | 16, 12 | Negative | 21.87 | 2 |
| 36 | Phloretin | 272.99 | 167^*^, 123 | 116 | 8, 20 | Negative | 22.30 | 2 |
| 37 | Kaempferol | 287.01 | 153^*^, 69.1 | 60 | 36, 50 | Positive | 23.84 | 2 |
| 38 | Isorhamnetin | 314.99 | 300.2^*^, 196.1 | 145 | 16, 4 | Negative | 24.57 | 2 |

^*^ These product ions were used for quantification.
